# Supplementary material for: Universal electrochemical quantification of active site density in transition metal nitrogen carbon electrocatalysts
Source: Nat Commun. 2025 Nov 27;16:10626. doi: 10.1038/s41467-025-65614-1 (PMC12660800; doi:10.1038/s41467-025-65614-1)
Supplement: Supplementary file 1 — Supplementary Information [file 41467_2025_65614_MOESM1_ESM.pdf]

Supplementary information for

Universal electrochemical quantification of active site density in transition metal nitrogen carbon electrocatalysts

Guang Li, Shu-Hu Yin,* Li-Fei Ji, Xu-Yuan Nie, Ting Zhu, Xiao-Yang Cheng, Jun Xu, Rui Huang, Yan-Xia Jiang,* Bin-Wei Zhang,* Shi-Gang Sun

Corresponding authors: shyin@ntu.edu.cn (Shu-Hu Yin); yxjiang@xmu.edu.cn (Yan-Xia Jiang); binwei@cqu.edu.cn (Bin-Wei Zhang)

Supplementary figures and tables

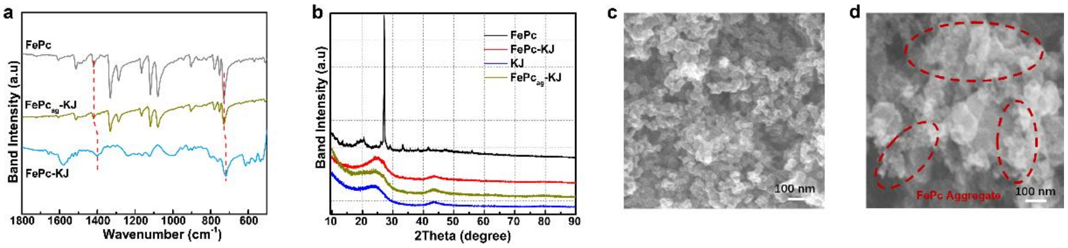

Supplementary Fig. 1 Structure characterization of FePc-KJ and FePc$_{ag}$-KJ. (a) Infrared spectra, (b) XRD pattern, (c)-(d) SEM image.

The absence of discernible FePc-derived XRD peaks in FePc$_{ag}$-KJ suggests agglomeration yielding crystallites below detection limits or disordered domains lacking coherent stacking. This distinction is corroborated by infraed spectroscopy: the preserved FePc fingerprint peaks in FePc$_{ag}$-KJ—closely matching pristine FePc—contrast sharply with the significantly altered peaks in FePc-KJ. This demonstrates that FePc$_{ag}$-KJ exhibits weak FePc-carrier interactions that permit agglomeration without electronic coupling sufficient to distort molecular vibrations, whereas FePc-KJ features strong interactions that modify vibrational modes, confirming carrier-induced amorphization.

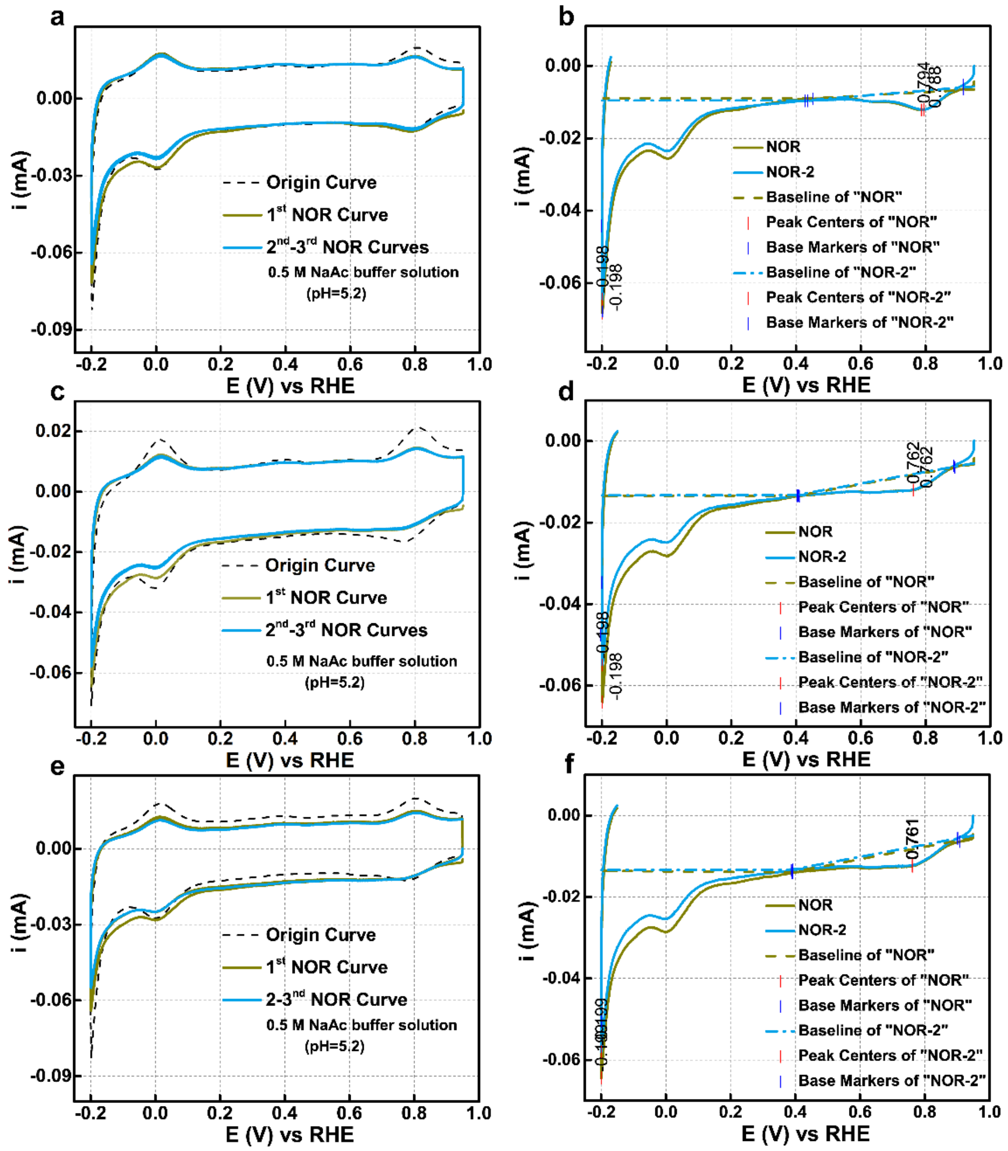

Supplementary Fig. 2 The NOR repeatability tests and integral electricity calculation of FePc-KJ by NPM method in the 0.5 M NaAc buffer solution (pH = 5.2). The resistance is about 20 Ω.

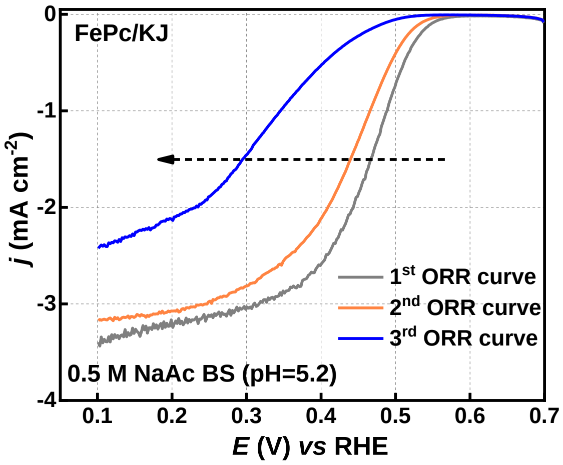

Supplementary Fig. 3 First three LSV curves of FePc-KJ at 900 rpm in O_2-saturated 0.5 M NaAc BS (pH = 5.2). The resistance is about 20 Ω.

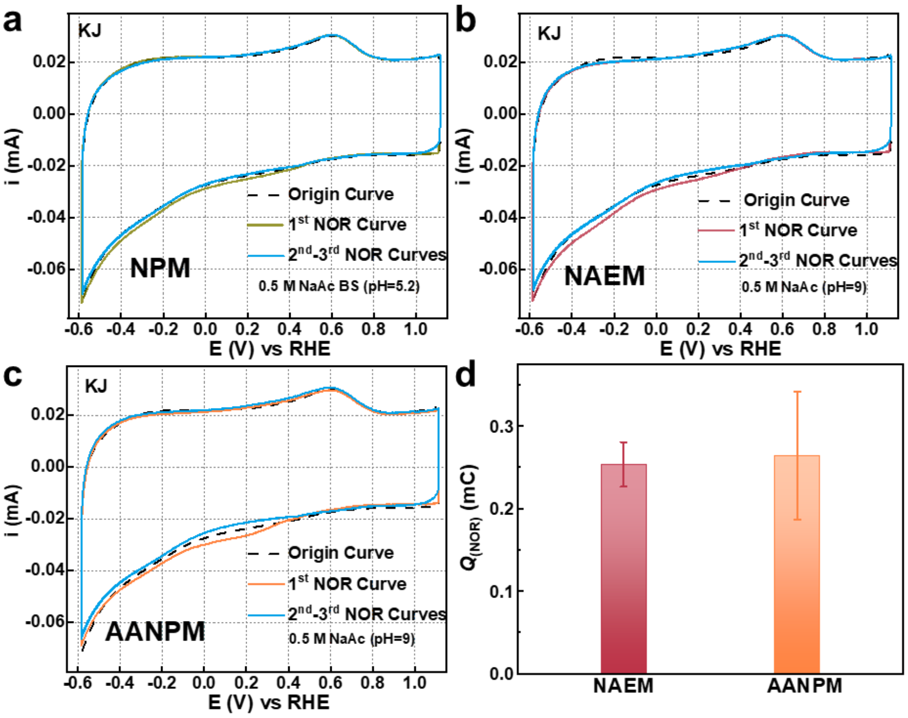

Supplementary Fig. 4 (a-c) NOR curves of FeNC with different methods; (d) Integral charge and poison effect of KJ. The resistance is about 20 Ω.

NOR tests on metal-free KJ carbon show negligible NOR activity (Q_{NOR} = 0.25 mC) and no NO-induced charge loss, further corroborating that NO's impact requires M-N_4 sites.

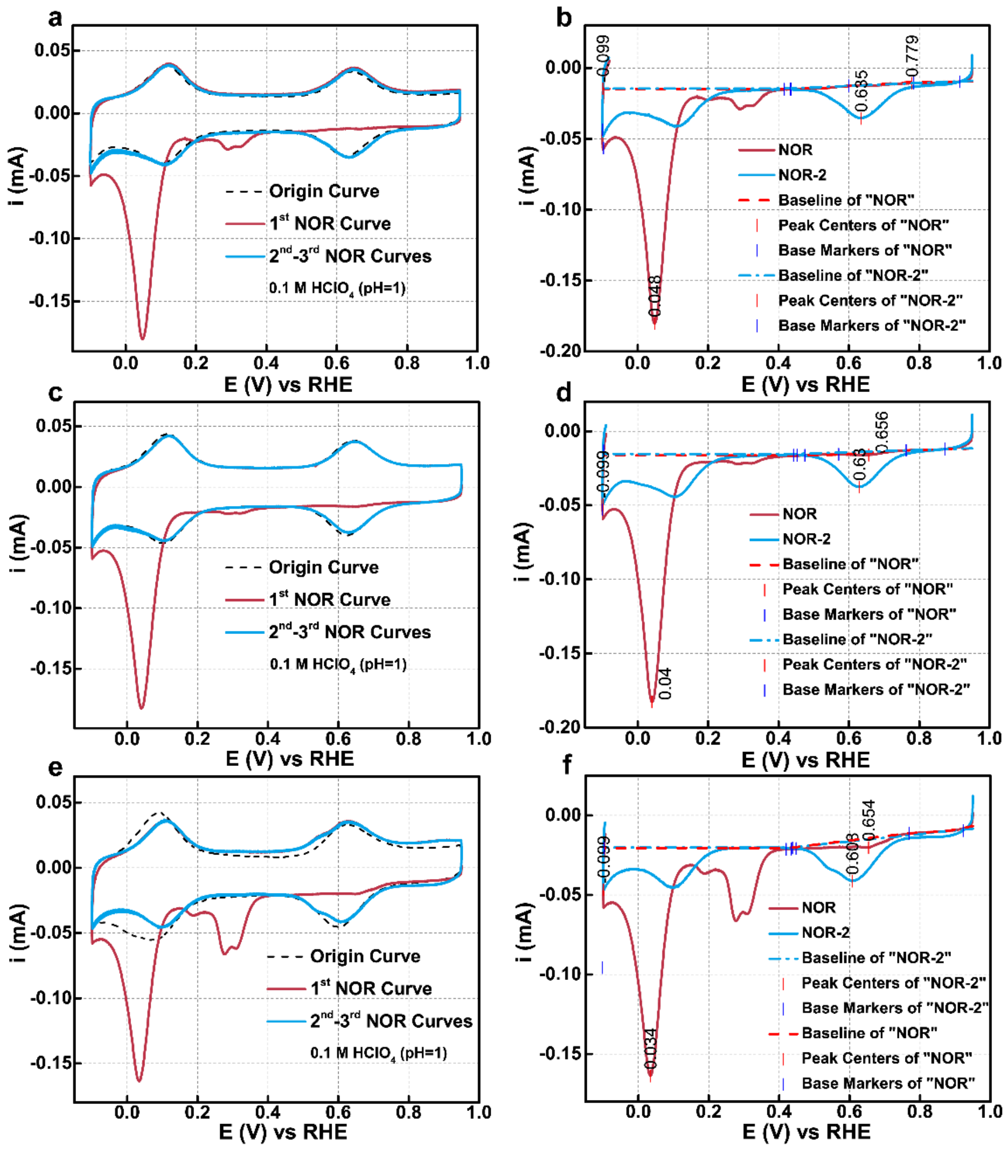

Supplementary Fig. 5 The NOR repeatability test and integral electricity calculation of FePc-KJ by NAEM method in the 0.1 M $HClO_4$ solution (pH = 1). The resistance is about 12 Ω.

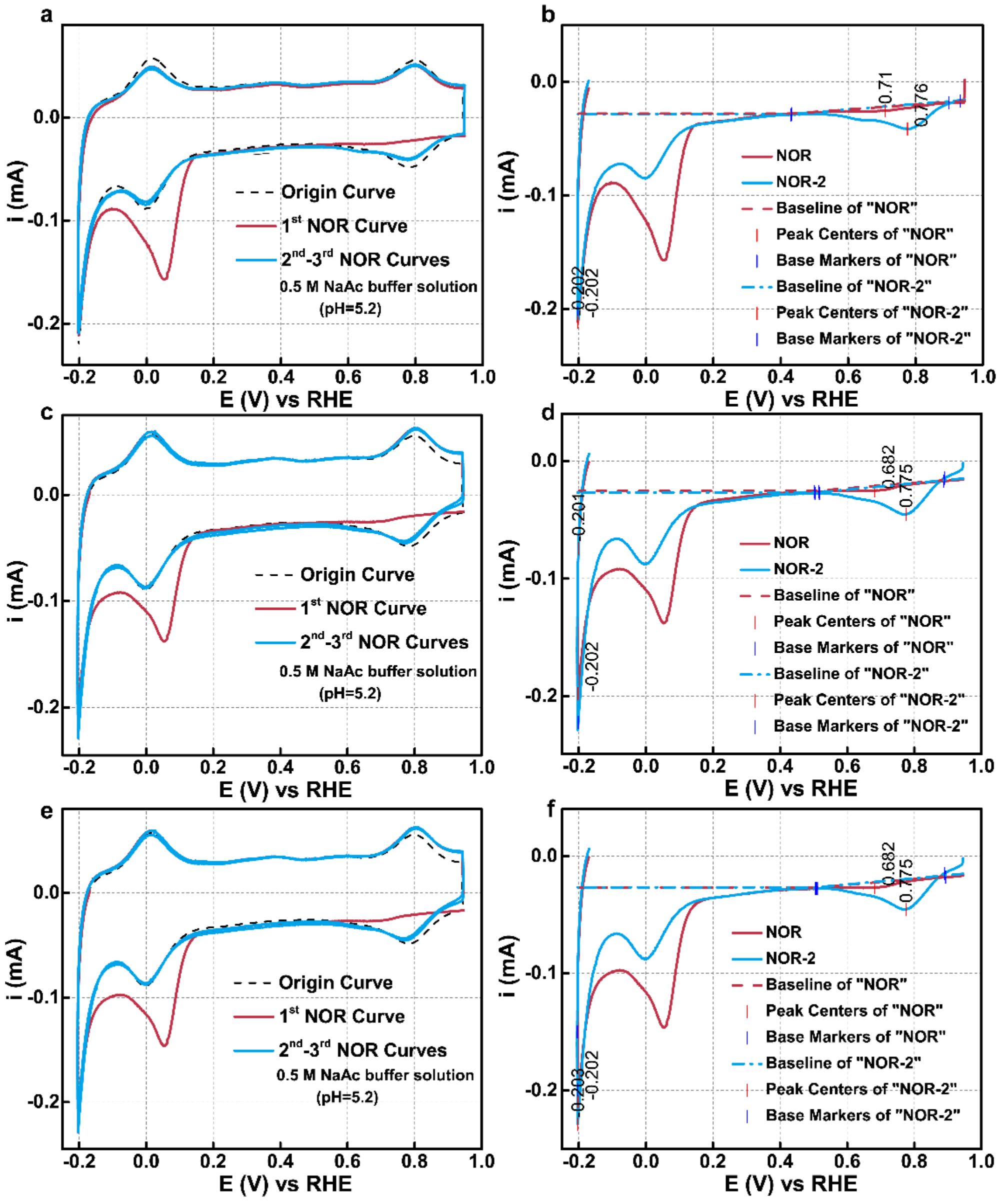

Supplementary Fig. 6 The NOR repeatability test and integral electricity calculation of FePc-KJ by NAEM method in the 0.1 M NaAc BS solution (pH = 5.2). The resistance is about 20 Ω.

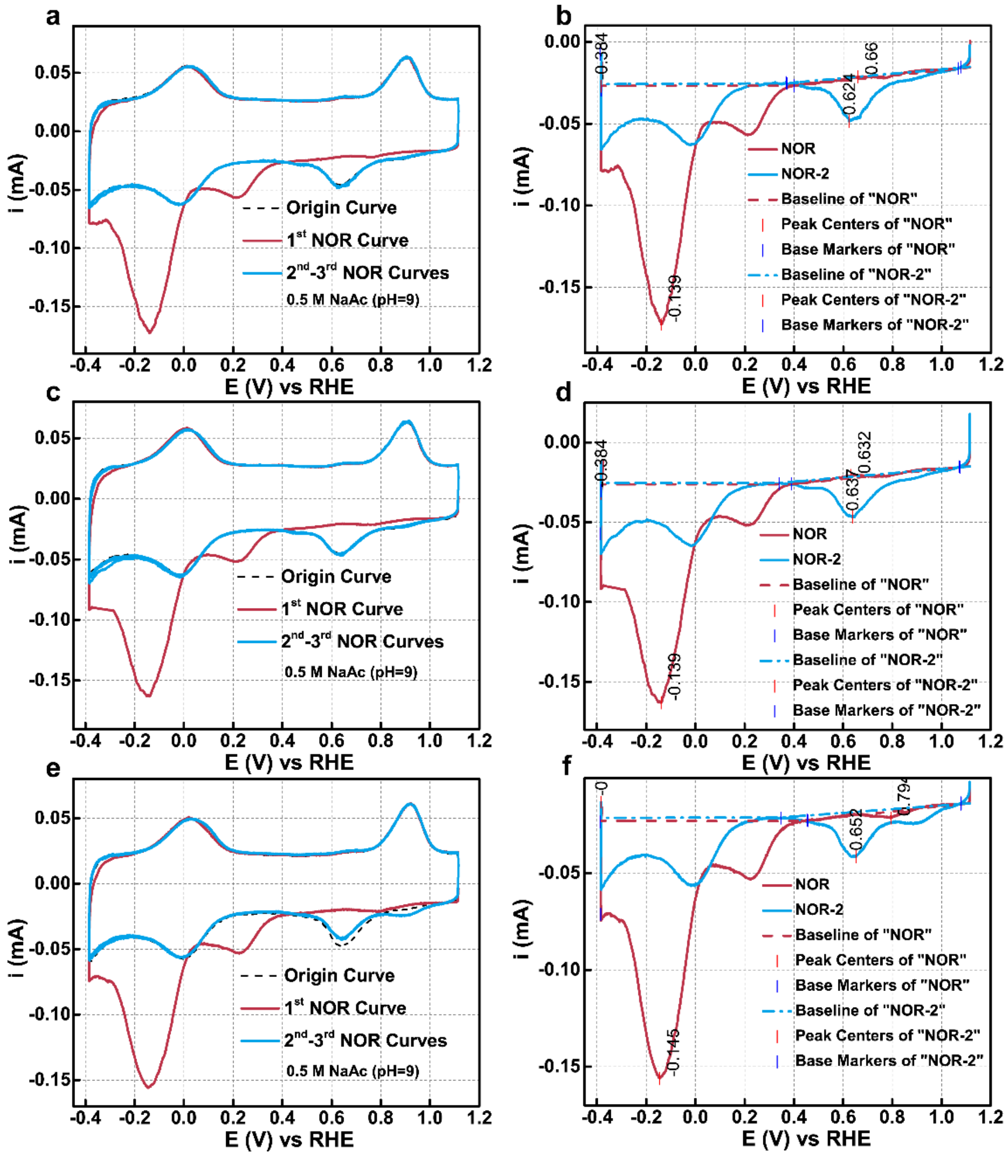

Supplementary Fig. 7 The NOR repeatability test and integral electricity calculation of FePc-KJ by NAEM method in the 0.5 M NaAc solution (pH = 9). The resistance is about 20 Ω.

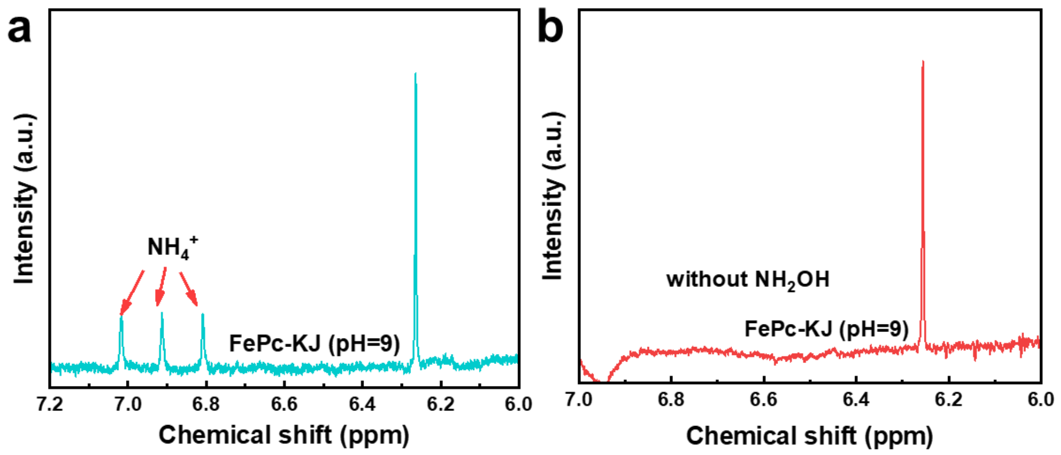

Supplementary Fig. 8 The NMR analysis for NOR products of FePc-KJ. (a) NH_4^+ spectra; (b) NH_2OH spectra. The *i-t* curve was tested at -0.38 V *vs.* RHE in NO-saturated 0.5 M NaAc solution (pH = 9).

NMR analysis indicated that NH_3 was the primary product formed during the 1-hour electrolysis of FePc-KJ in NaAc solution (pH = 9), with NH_2OH virtually undetectable. This demonstrated that NO was predominantly reduced to NH_3 via a $5e^-$ pathway at pH = 9, consistent with the quantitative charge analysis results.

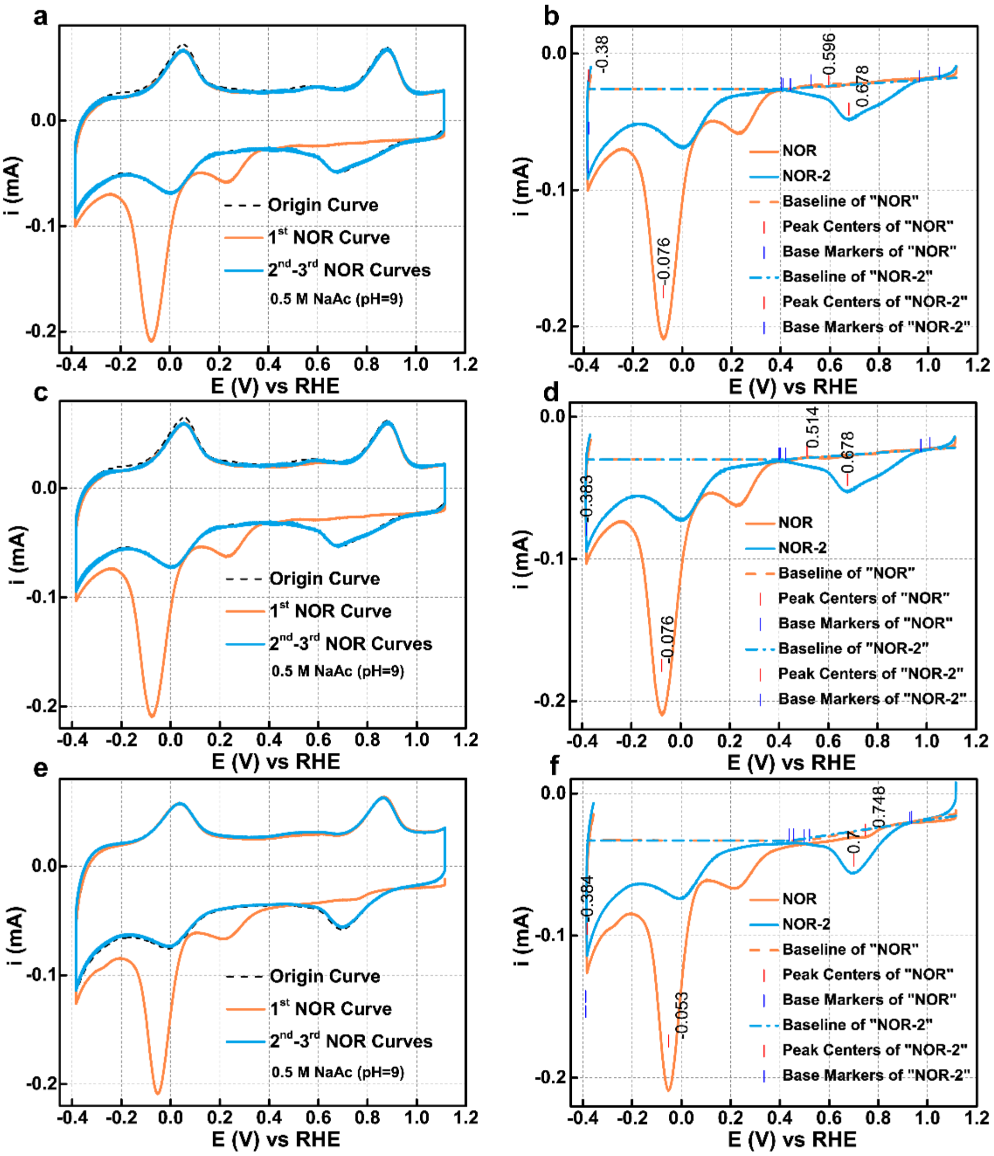

Supplementary Fig. 9 The NOR repeatability tests and integral electricity calculation of FePc-KJ by AANPM method in the 0.5 M NaAc solution (pH = 9). The resistance is about 20 Ω.

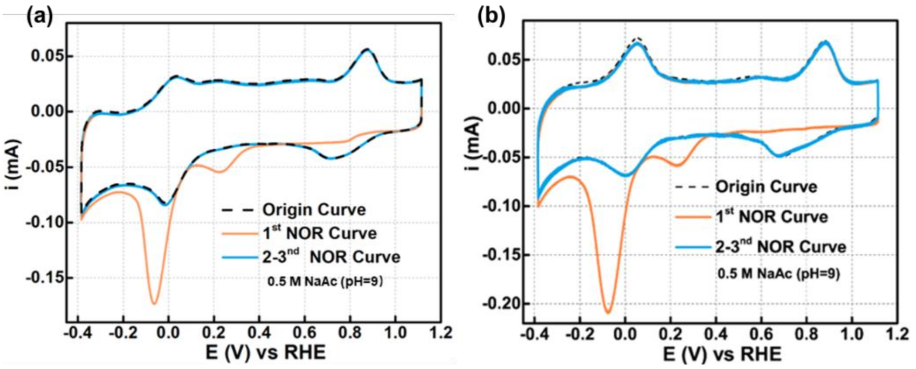

Supplementary Fig. 10 NOR curves of FePc-KJ catalyst by AANPM in different pH solutions. (a) 0.001 M $HClO_4$ + 0.125 M $NaNO_2$ solution (pH = 3); (b) 0.01 M $HClO_4$ + 0.125 M $NaNO_2$ solution (pH = 2). The resistance is about 15 Ω.

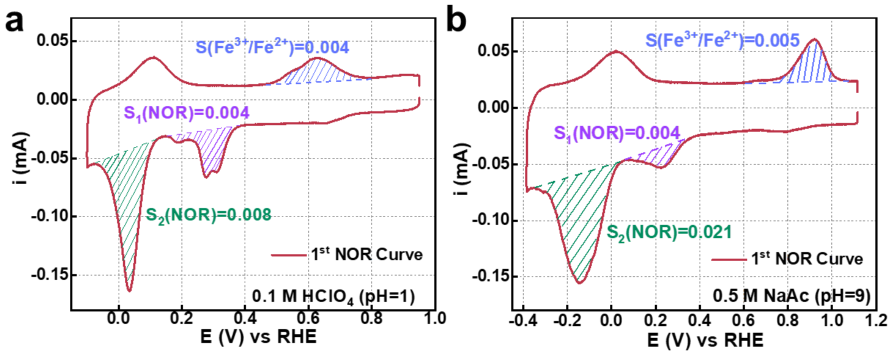

Supplementary Fig. 11 The integrated charge of Fe^{3+}/Fe^{2+} oxidation peak and NOR reduction peak at (a) pH = 1 and (b) pH =9.

The additional reduction peaks observed at approximately 0.3 V (pH = 1) and 0.2 V (pH = 9) are correspond to the initial one-electron reduction step of NO to nitroxyl (HNO) within the NOR process. This assignment is strongly supported by quantitative charge analysis, which demonstrates that the integrated charge under both peaks equates to a single electron transfer (1× ratio) relative to the Fe^{3+}/Fe^{2+} redox couple.

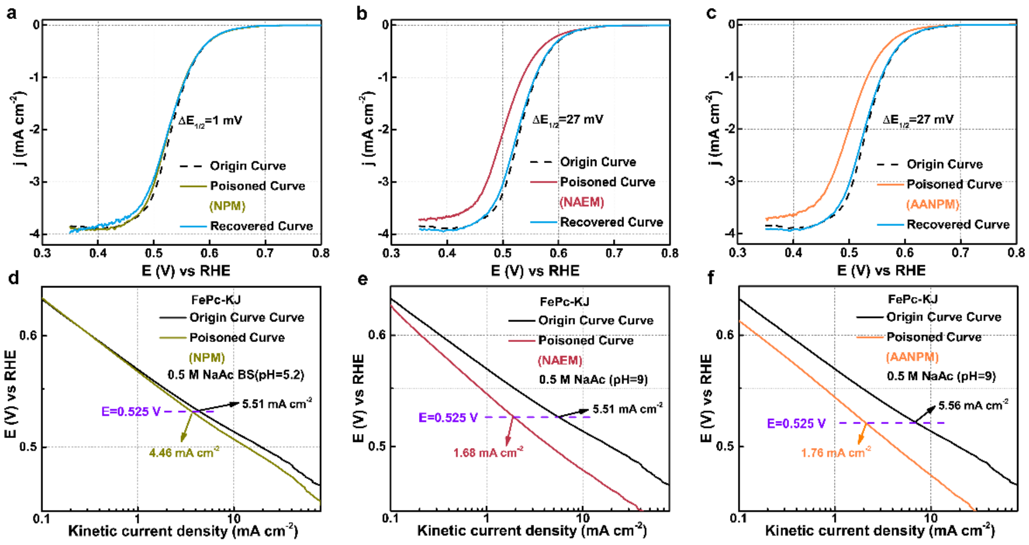

Supplementary Fig. 12 The ORR test and calculated kinetic current in the origin condition, poisoned condition and recovered condition of FePc-KJ in NPM, NAEM and AANPM processes. The resistance is about 20 Ω.

The inferior recovered ORR activity in acidic NPM arises from the inherent limitations of NO poisoning in FePc systems and acid-induced catalyst degradation. In alkaline AANPM/NAEM, the near-complete recovery of activity confirms the method's precision and the stability of FePc under alkaline conditions.

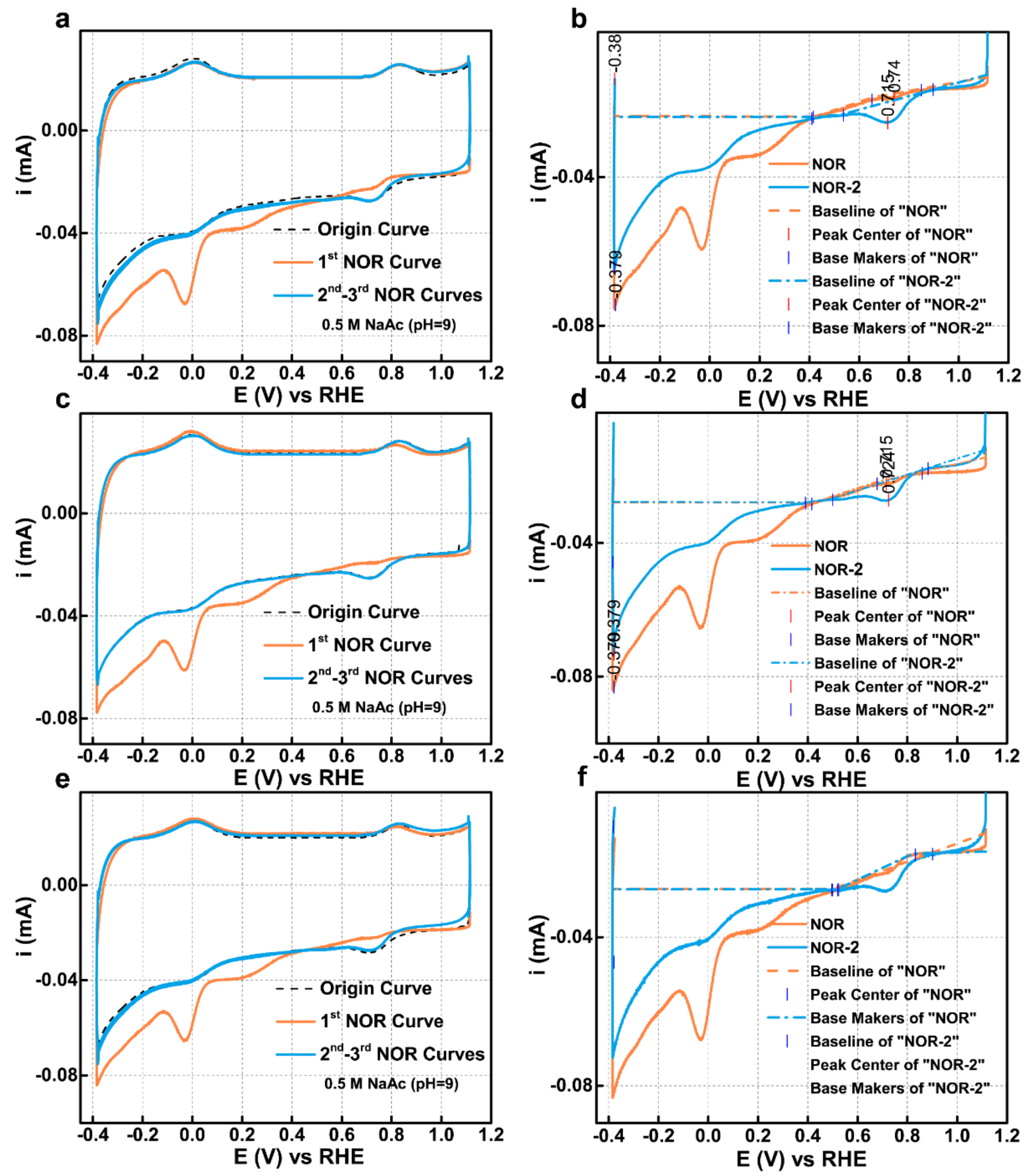

Supplementary Fig. 13 The NOR repeatability test and integral electricity calculation of $FePc_{ag}$-KJ by AANPM method in the 0.5 M NaAc solution (pH = 9). The resistance is about 20 Ω.

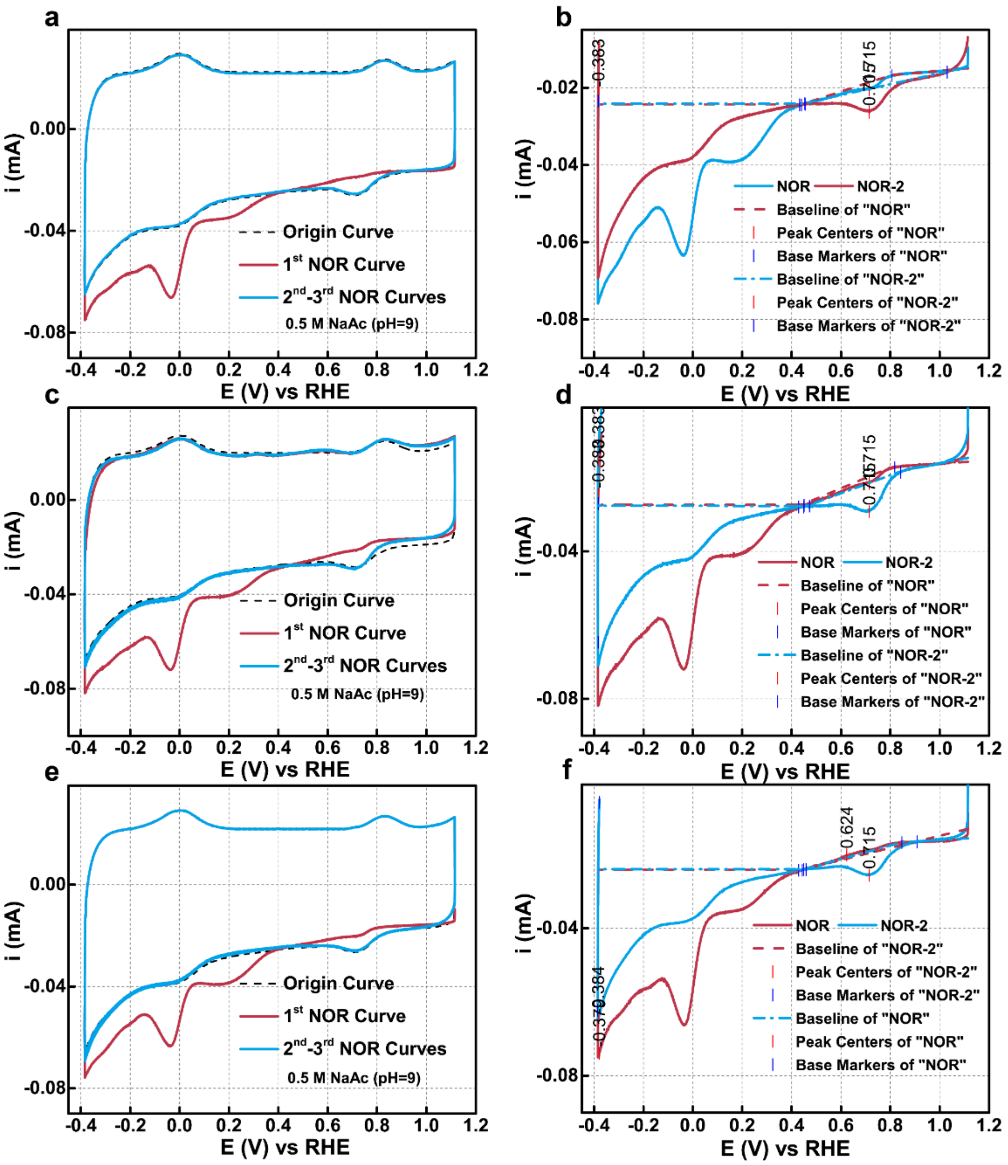

Supplementary Fig. 14 The NOR repeatability test and integral electricity calculation of FePc$_{ag}$-KJ by NAEM method in the 0.5 M NaAc solution (pH = 9). The resistance is about 20 Ω.

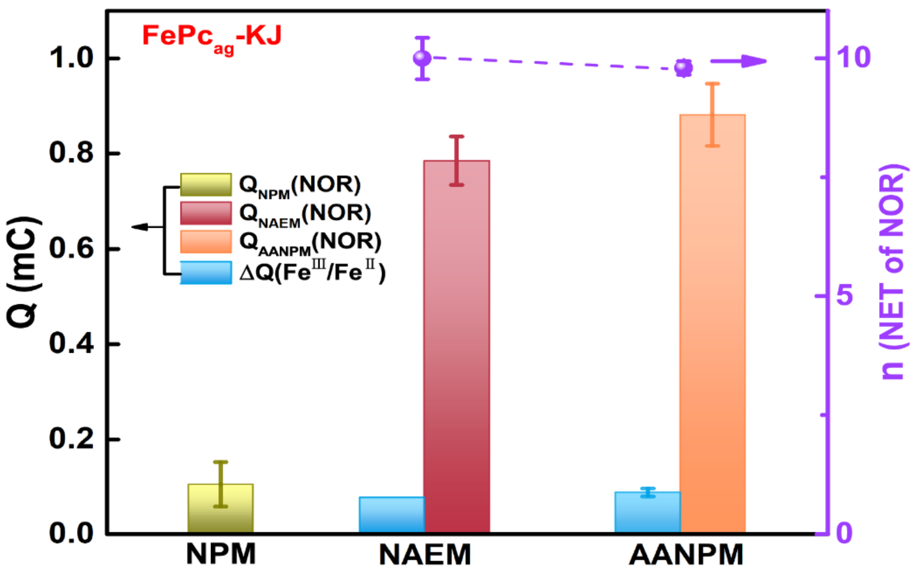

Supplementary Fig. 15 The integrated charge and electron transfer numbers (n) of FePc$_{ag}$-KJ catalyst in different methods.

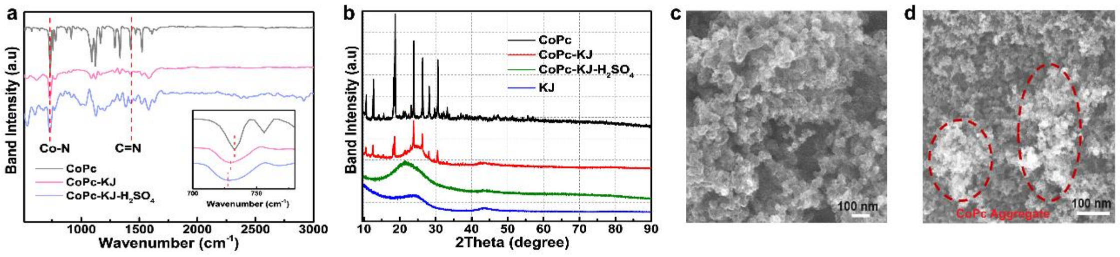

Supplementary Fig. 16 Structure characterization of CoPc$_{ag}$-KJ and CoPc-KJ catalysts. (a) Infrared spectra; (b) XRD pattern; (c)-(d) SEM images.

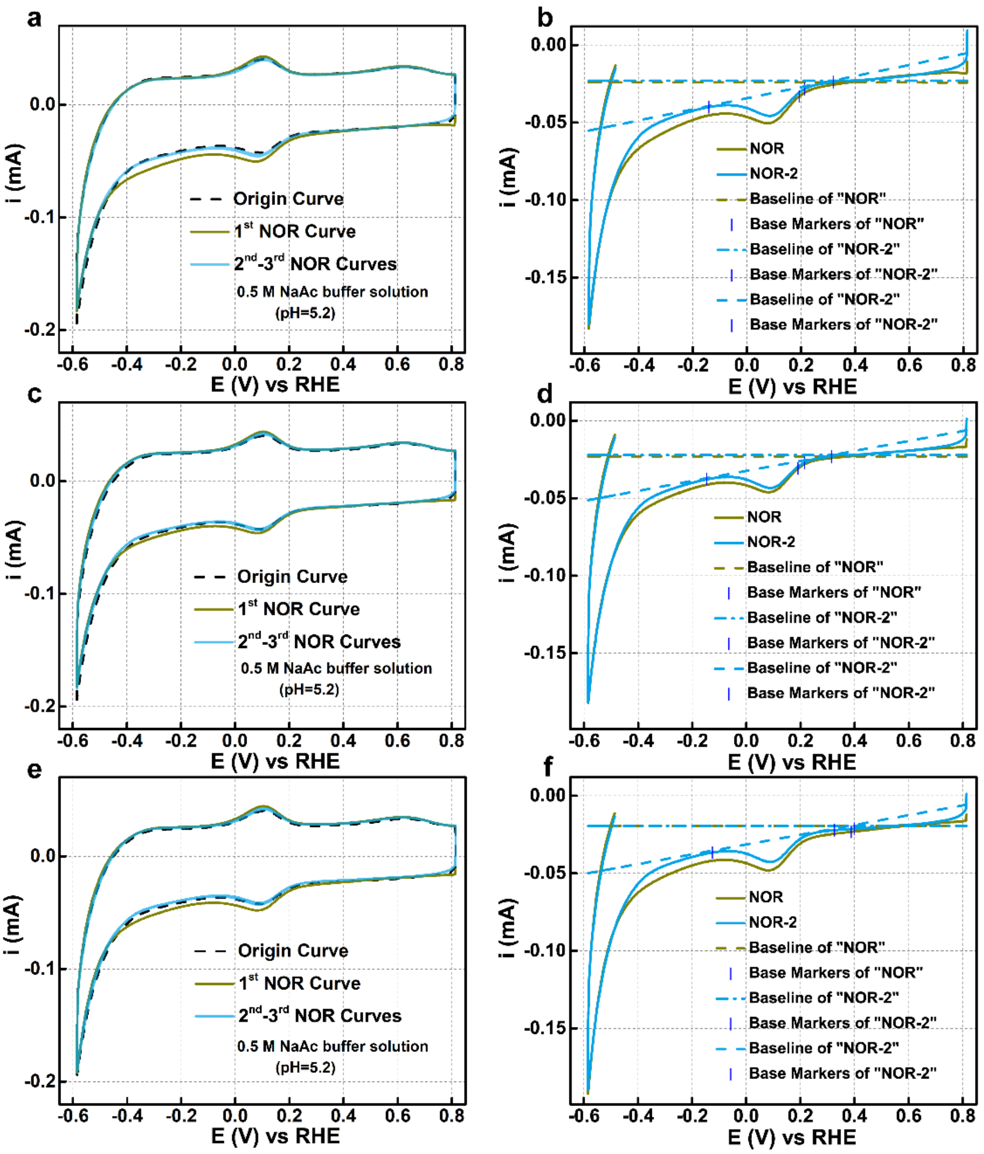

Supplementary Fig. 17 The NOR repeatability test and integral electricity calculation of $CoPc_{ag}$-KJ by NPM method in the 0.5 M NaAc buffer solution (pH = 5.2). The resistance is about 20 Ω.

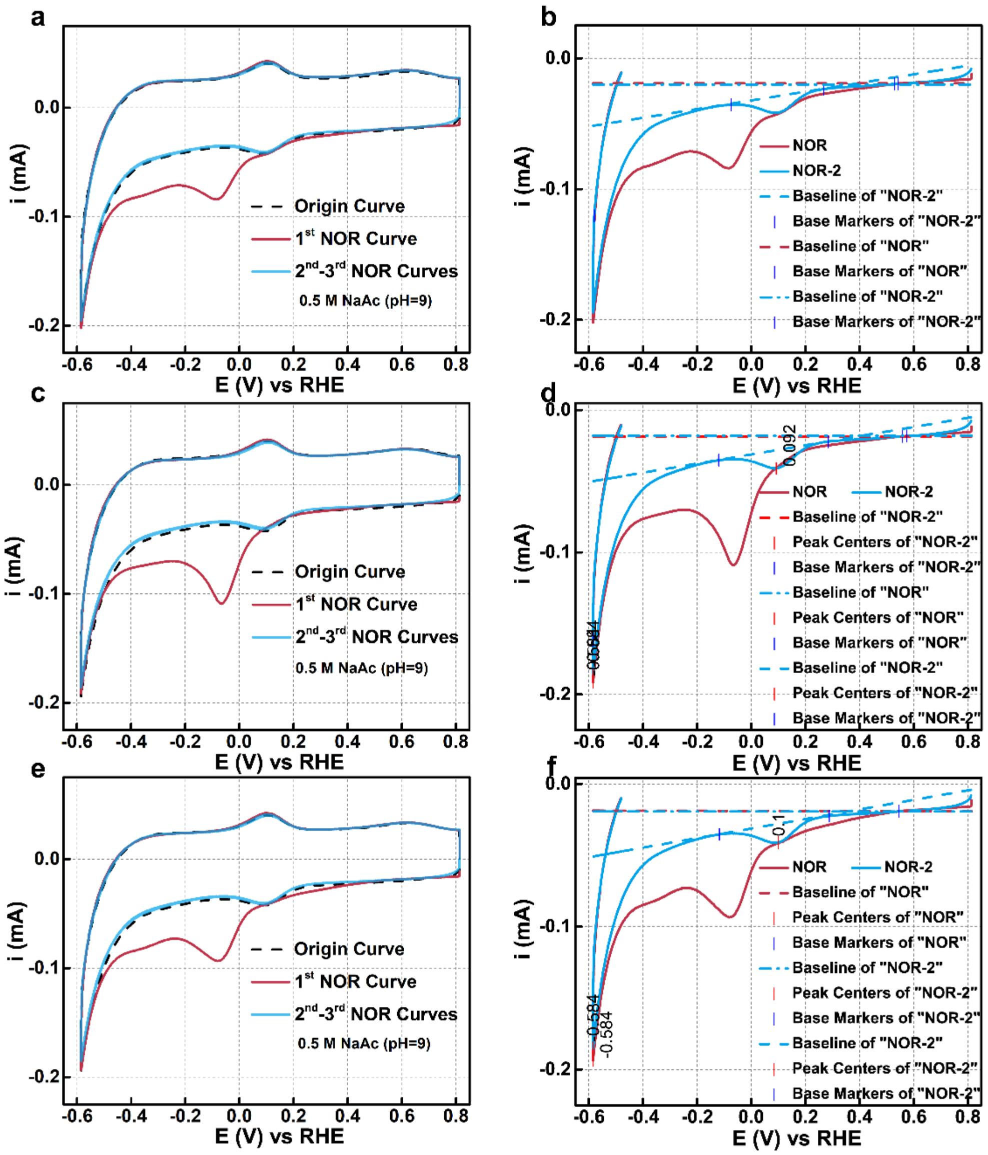

Supplementary Fig. 18 The NOR repeatability test and integral electricity calculation of CoPc$_{ag}$-KJ by NAEM method in the 0.5 M NaAc solution (pH = 9). The resistance is about 20 Ω.

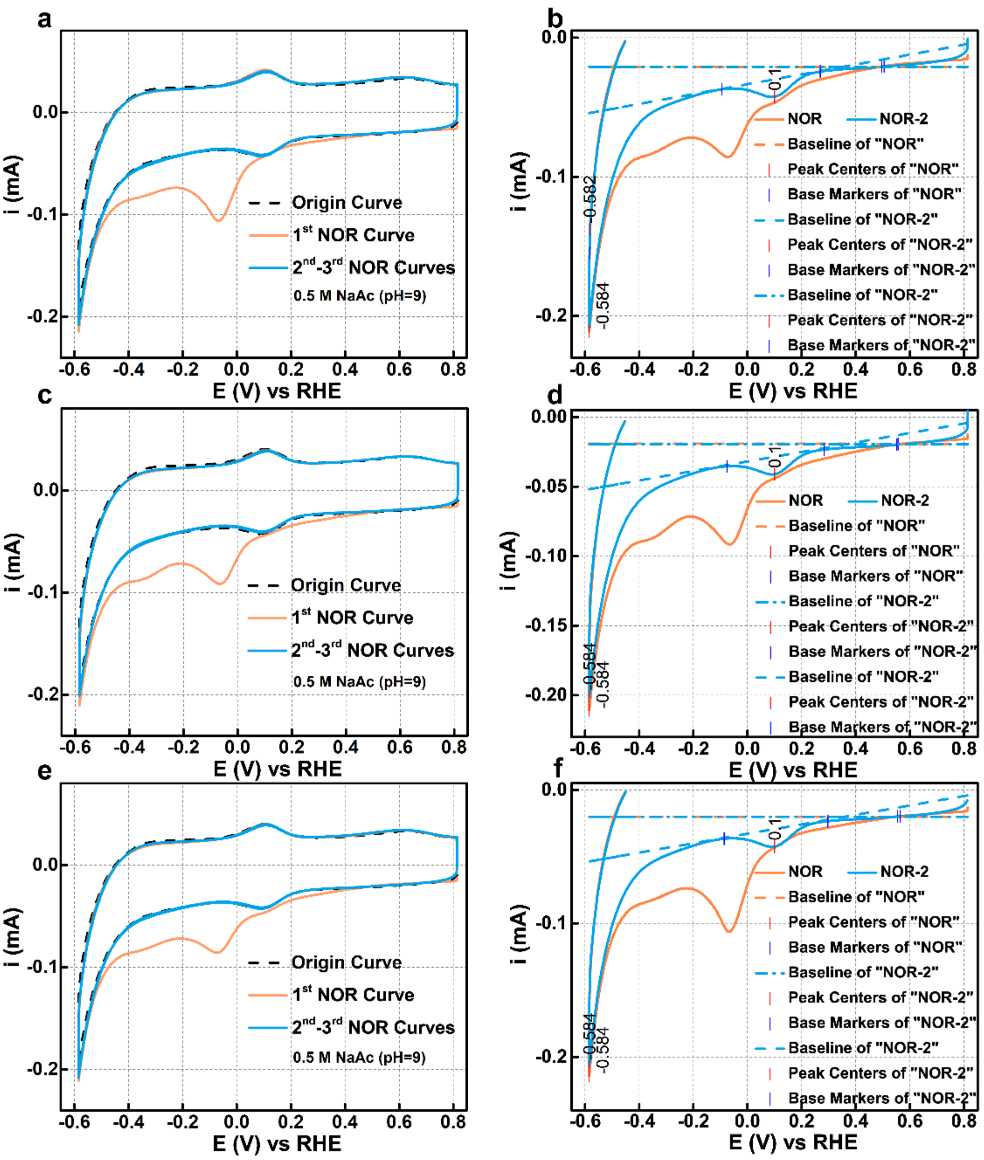

Supplementary Fig. 19 The NOR repeatability test and integral electricity calculation of $CoPc_{ag}$-KJ by AANPM method in the 0.5 M NaAc solution (pH = 9). The resistance is about 20 Ω.

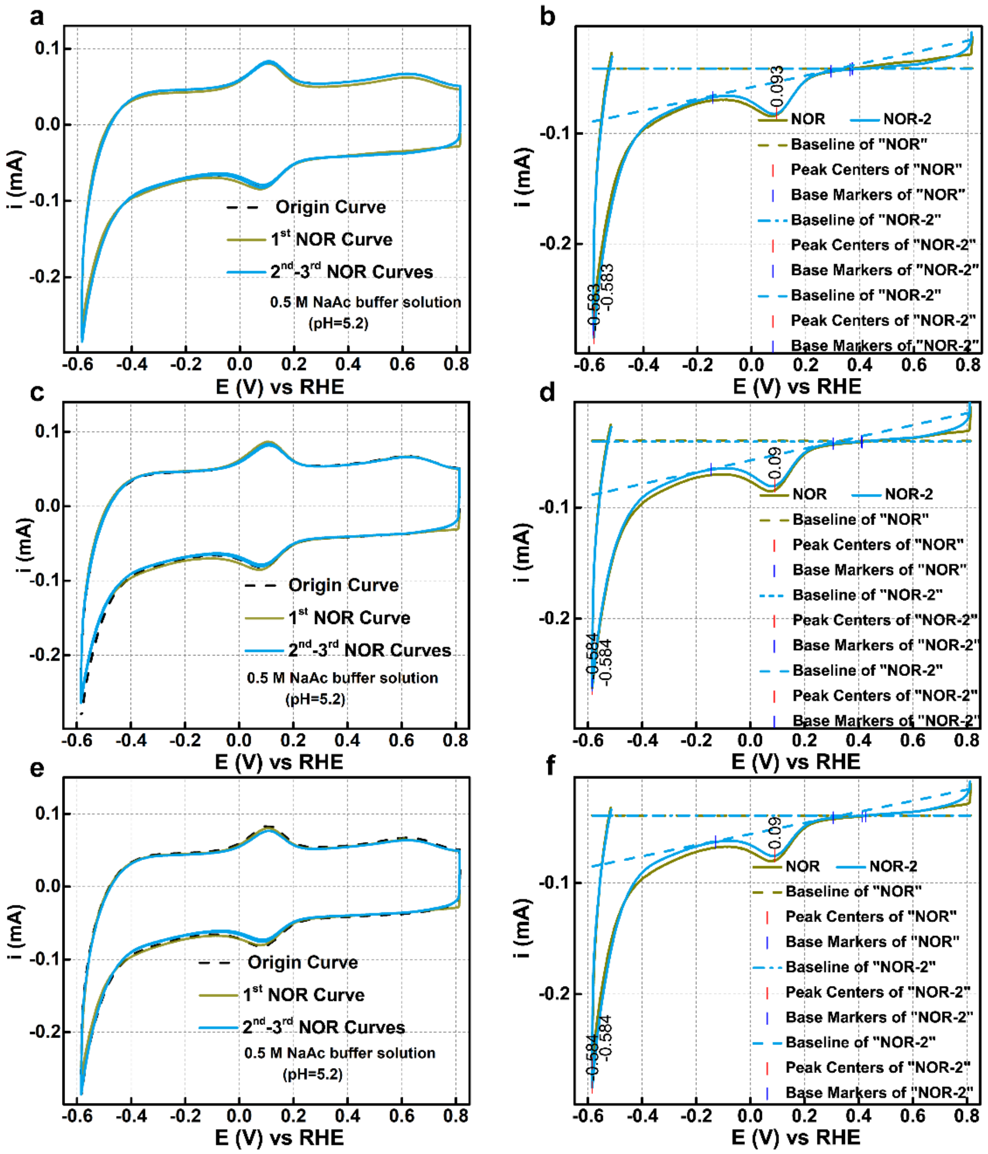

Supplementary Fig. 20 The NOR repeatability test and integral electricity calculation of CoPc-KJ by NPM method in the 0.5 M NaAc buffer solution (pH = 5.2). The resistance is about 20 Ω.

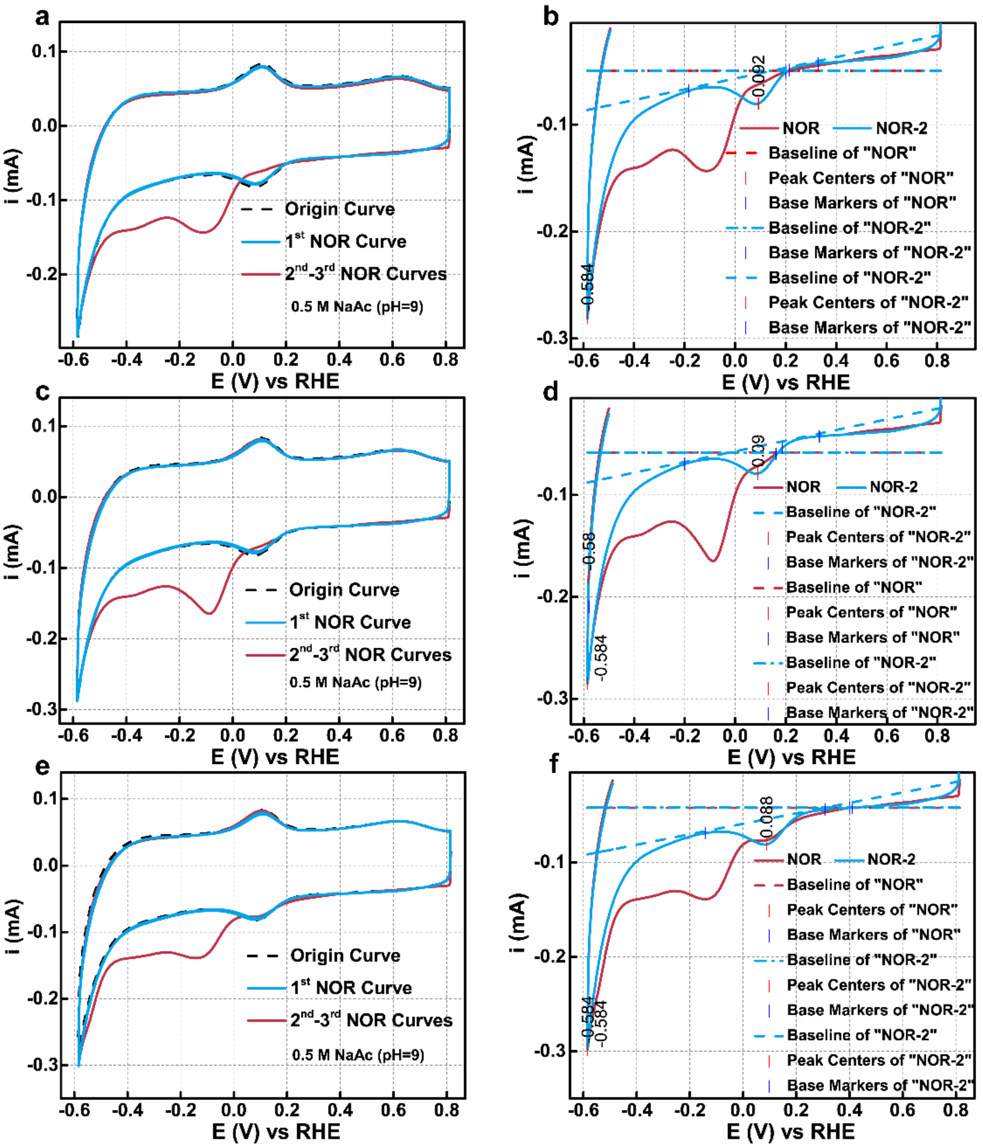

Supplementary Fig. 21 The NOR repeatability test and integral electricity calculation of CoPc-KJ by NAEM method in the 0.5 M NaAc solution (pH = 9). The resistance is about 20 Ω.

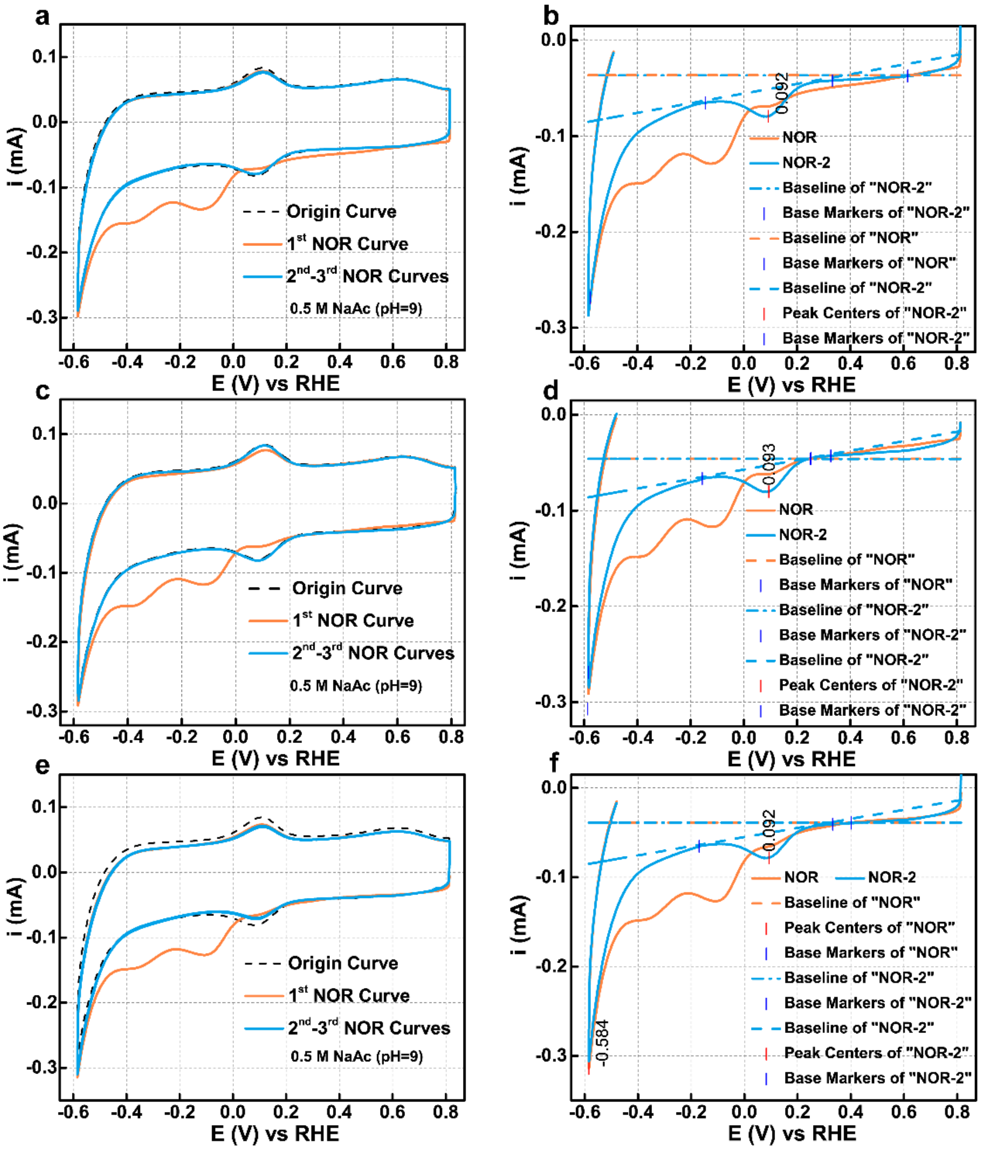

Supplementary Fig. 22 The NOR repeatability test and integral electricity calculation of CoPc-KJ by AANPM method in the 0.5 M NaAc solution (pH = 9). The resistance is about 20 Ω.

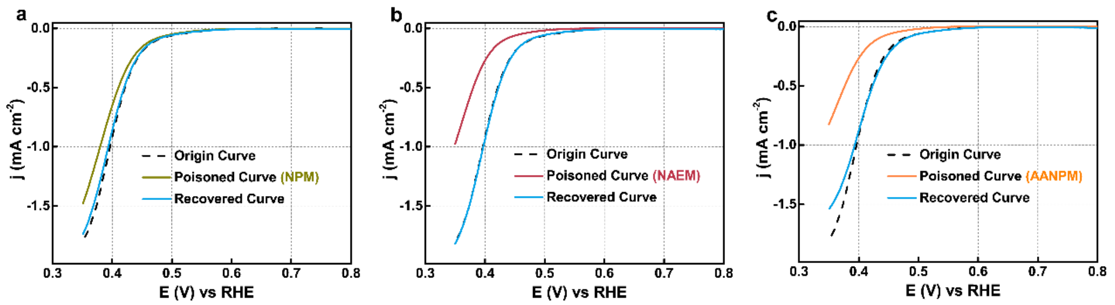

Supplementary Fig. 23 The ORR test in the origin condition, poisoned condition and recovered condition of $CoPc_{ag}$-KJ with different poisoned method. The resistance is about 20 Ω.

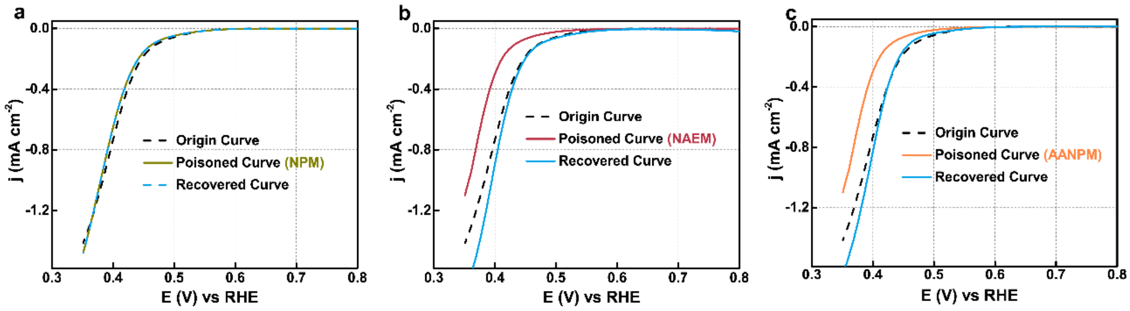

Supplementary Fig. 24 The ORR test in the origin condition, poisoned condition and recovered condition of CoPc-KJ with different poisoned method. The resistance is about 20 Ω.

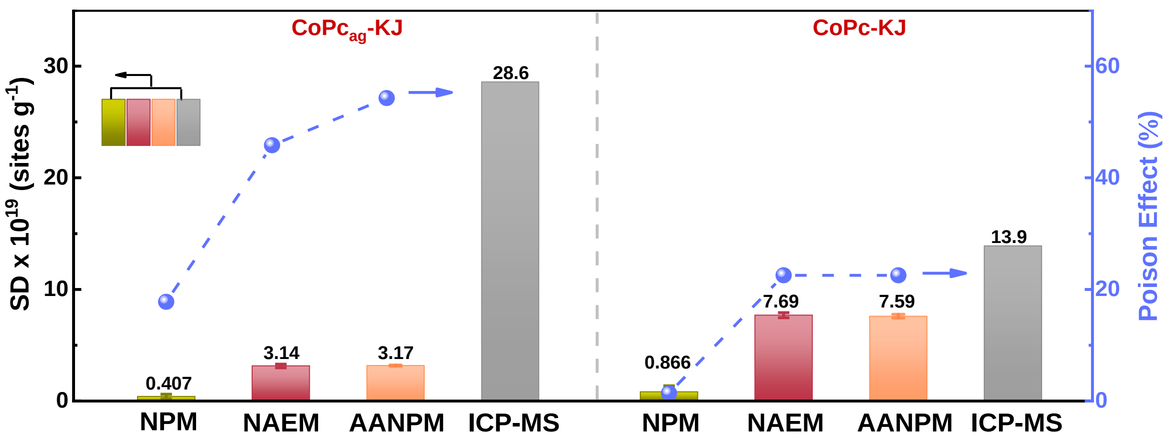

Supplementary Fig. 25 The SD and poisoning effect of CoPc$_{ag}$-KJ and CoPc-KJ catalysts in different methods.

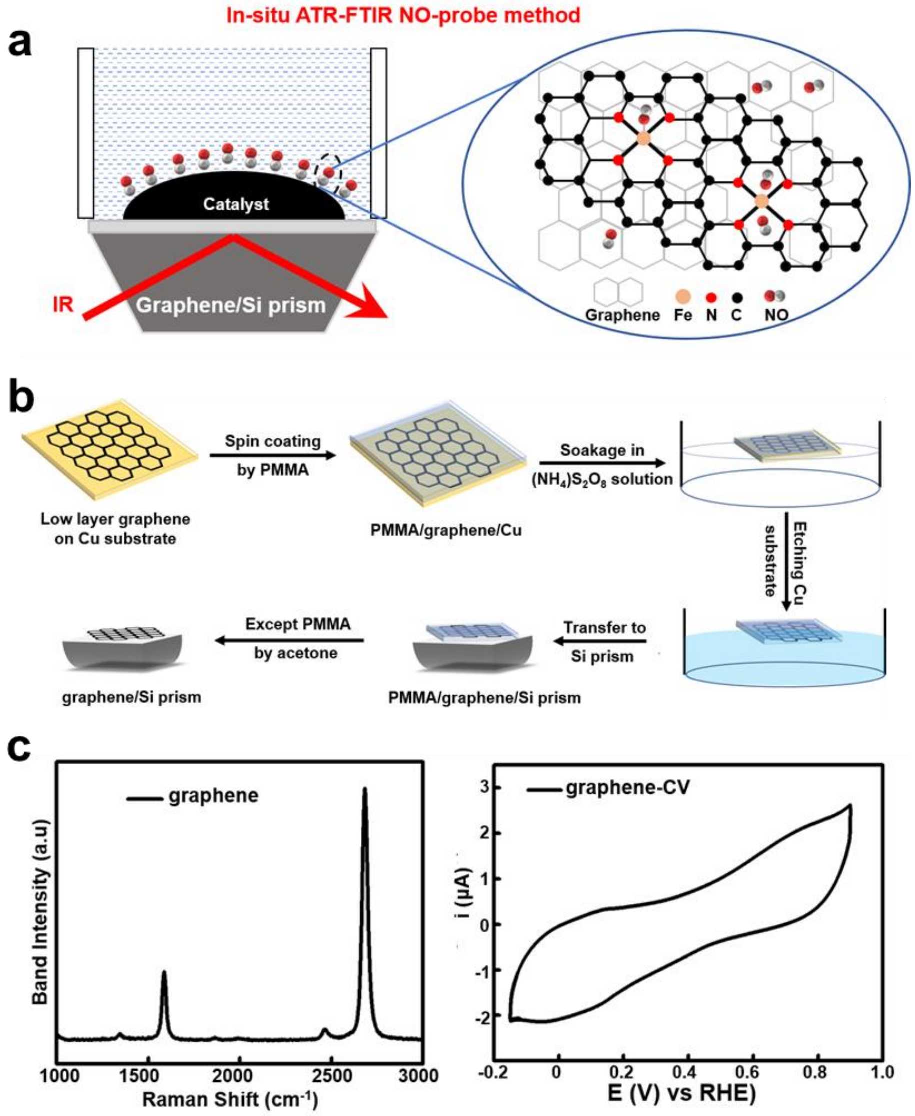

Supplementary Fig. 26 Gra-based *in-situ* ATR-FTIR and analysis of NO adsorption process. (a) Schematic diagram of gra-based *in-situ* ATR-FTIR and NO adsorption; (b) Low-layer graphene transfer process; (c) Raman spectra of graphene/Si prism.

The structure characterization and electrochemistry test of graphene/Si prism were shown in **Fig. 26c**. The shape peak at 2700 cm^{-1} in the Raman spectra could be assigned as 2D peak of graphene; the small peak at 1350 cm^{-1} meant the few defects in the graphene. The cyclic voltammetry curve of graphene/Si prism demonstrated the electrochemical stability of graphene even at high potential (0.9 V).

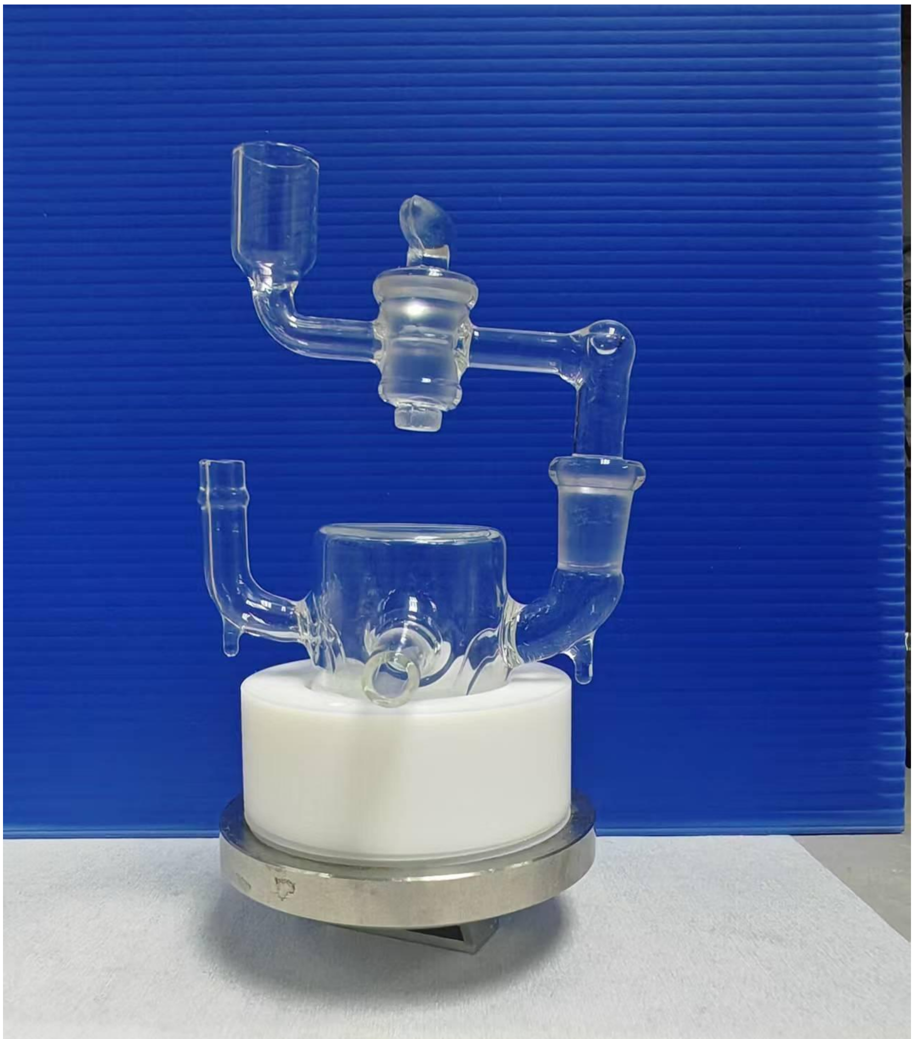

Supplementary Fig. 27 The custom-built electrochemical cells for ATR-FTIR.

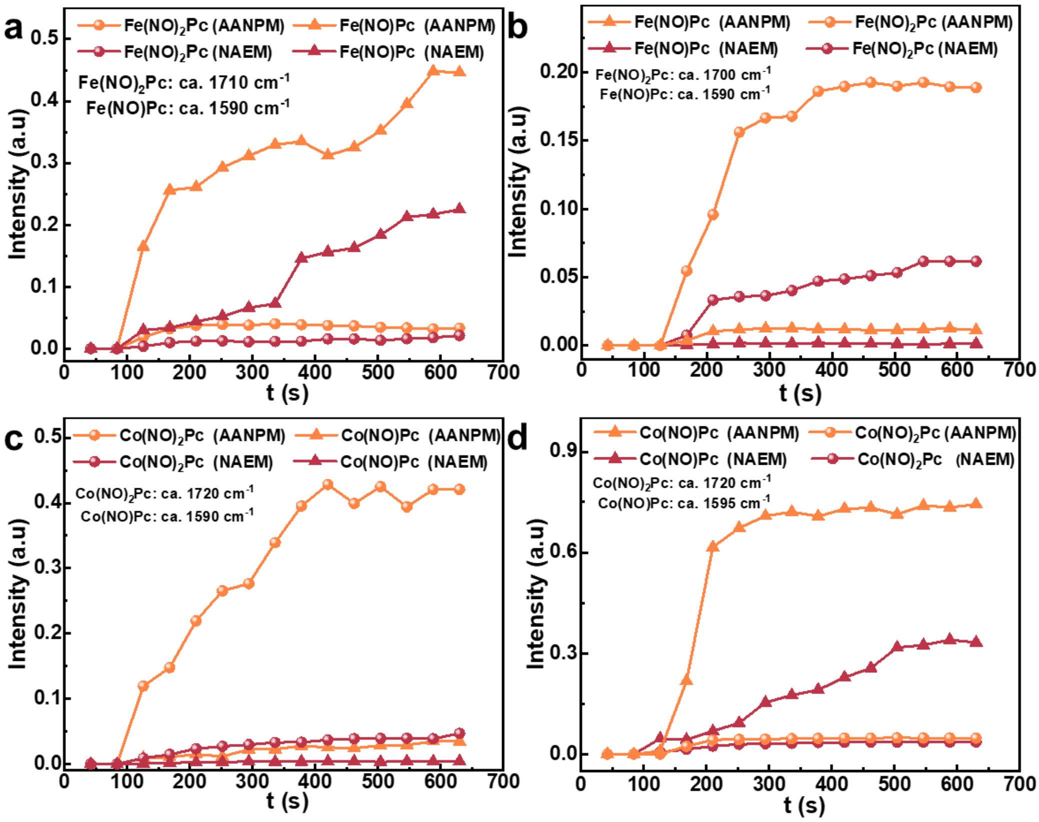

Supplementary Fig. 28 The changes of NO peak intensity in the NAEM and ANPM (a) FePc-KJ (b) FePc$_{ag}$-KJ (c) CoPc$_{ag}$-KJ (d) CoPc-KJ.

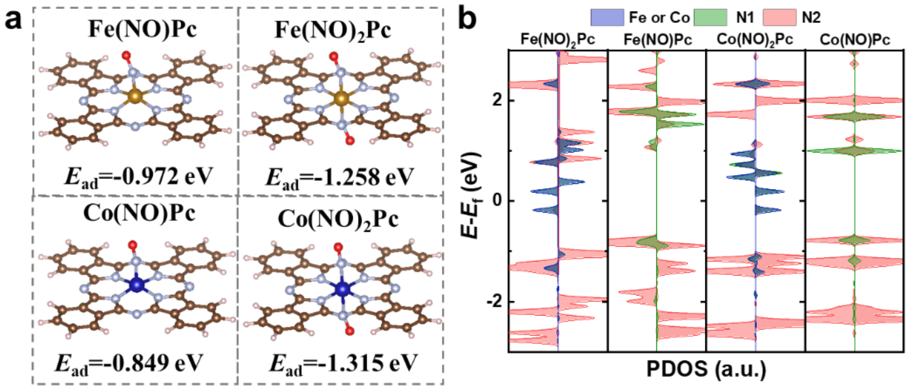

Supplementary Fig. 29. (a) The calculated adsorption energies for M(NO)Pc and $M(NO)_2Pc$. (d) Projected spin-resolved density of states (PDOS) diagram of Fe/Co and N in $M(NO)_2Pc$.

NO preferentially adsorbs onto metal centers (Fe/Co) in FePc/CoPc via adsorbing one NO molecule and two NO molecules, with adsorption energies of -0.972 eV and -1.258 eV for FePc (-0.849 eV and -1.315 eV for CoPc), respectively. Projected density of states (PDOS) reveals strong hybridization between Fe/Co *d*-orbitals and NO π orbitals, underscoring selective metal binding.

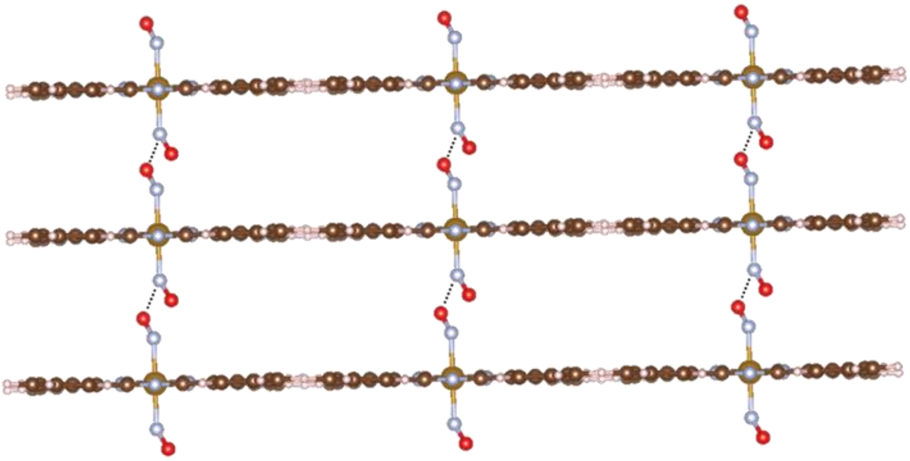

Supplementary Fig. 30 The possible model structure of dual-NO adsorption via *μ*-(N,O) bridges (Fe-N≡O···N≡O-Fe).

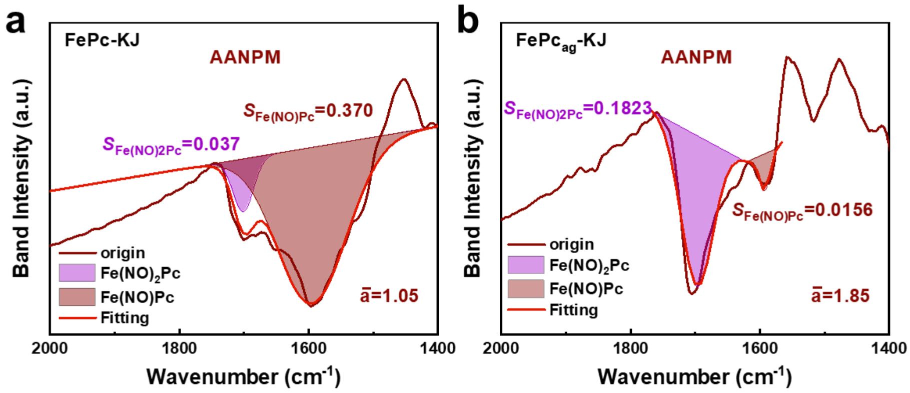

Supplementary Fig. 31 NO_{ad} spectra and the corresponding spectral peak intergral area of $S_{Fe(NO)2Pc}$ and $S_{Fe(NO)Pc}$ in AANPM process for (a) FePc-KJ and (b) $FePc_{ag}$-KJ.

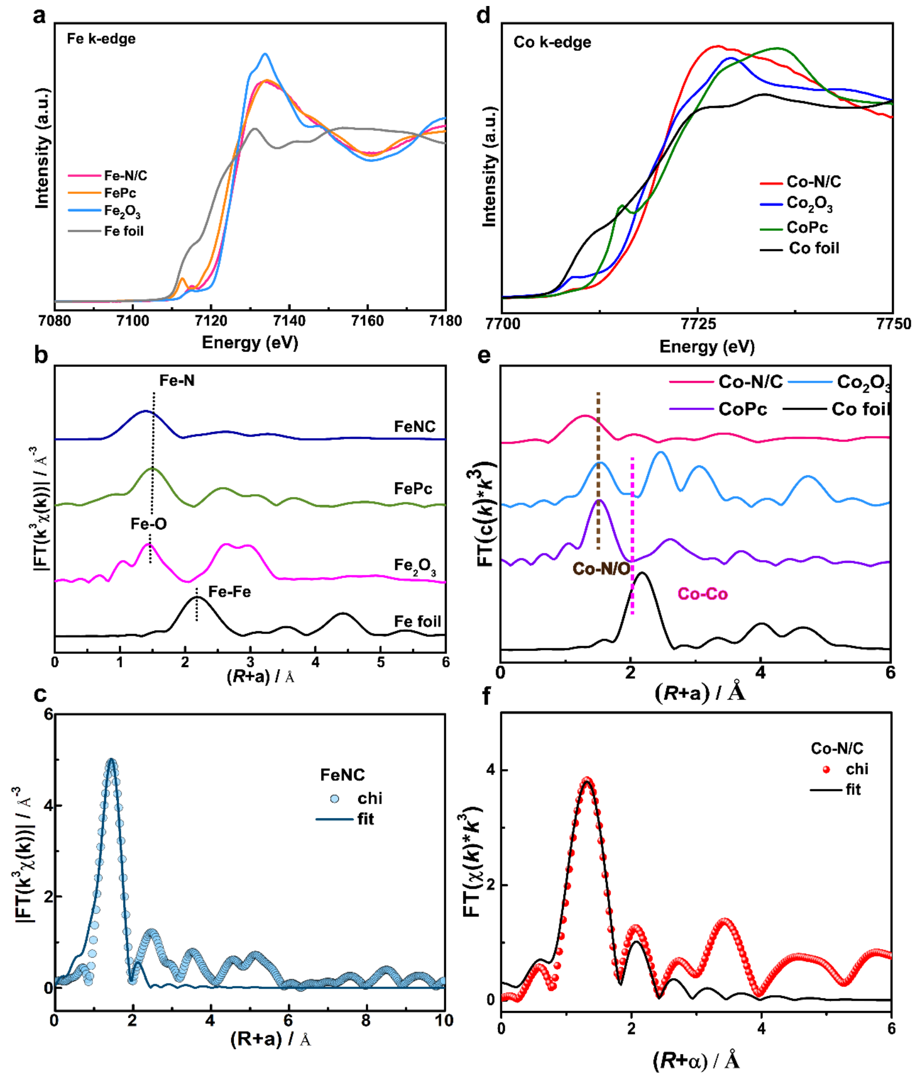

Supplementary Fig. 32 Atomic structural analysis of FeNC and CoNC. (a) Fe K-edge XANES spectra; (b) Fe K-edge FT-EXAFS spectra; (c) Fe K-edge EXAFS fitting analysis in R space; (d) Fe K-edge XANES spectra; (e) Fe K-edge FT-EXAFS spectra; (f) Fe K-edge EXAFS fitting analysis in R space.

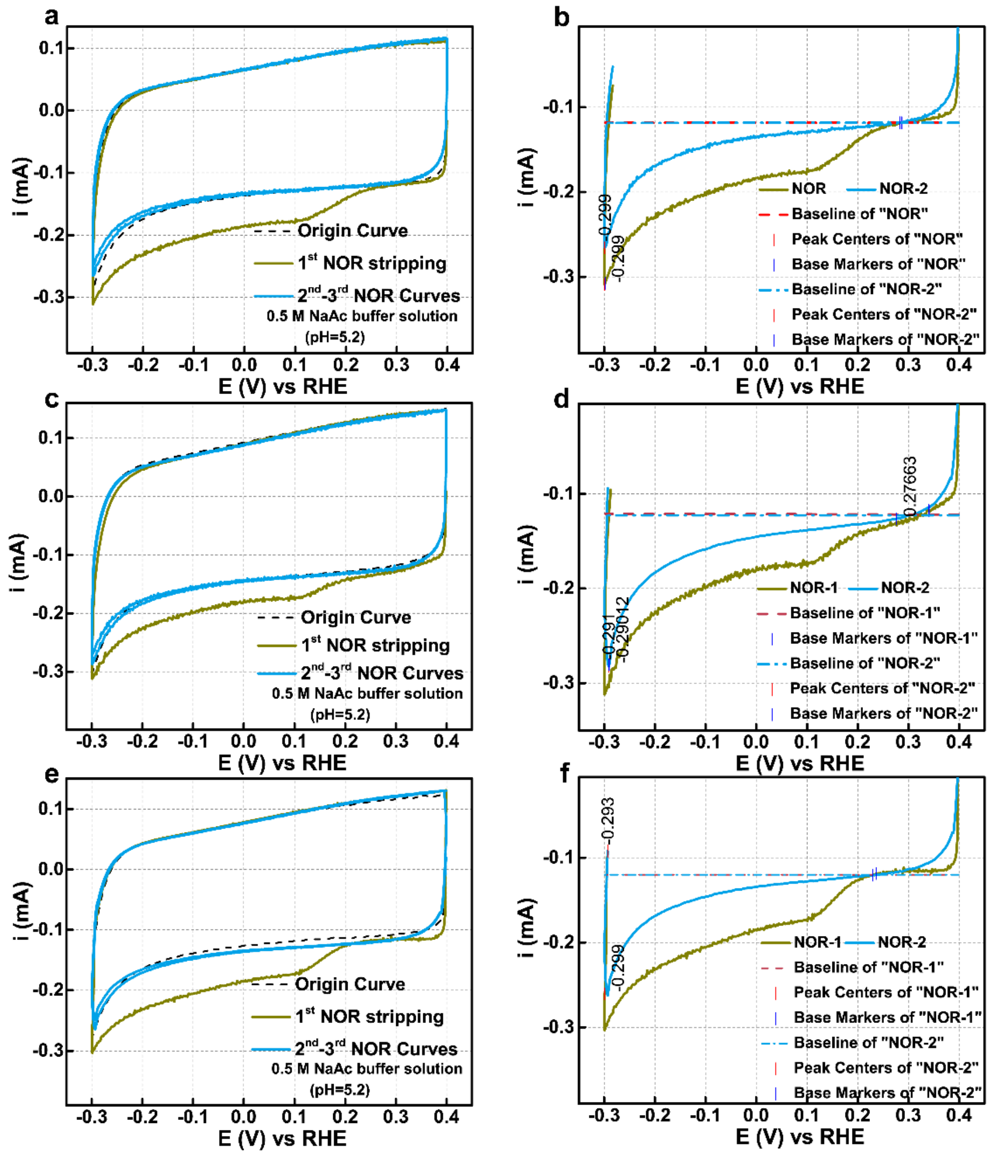

Supplementary Fig. 33 The NOR repeatability test and integral electricity calculation of FeNC by NPM method in the 0.5 M NaAc buffer solution (pH = 5.2). The resistance is about 20 Ω.

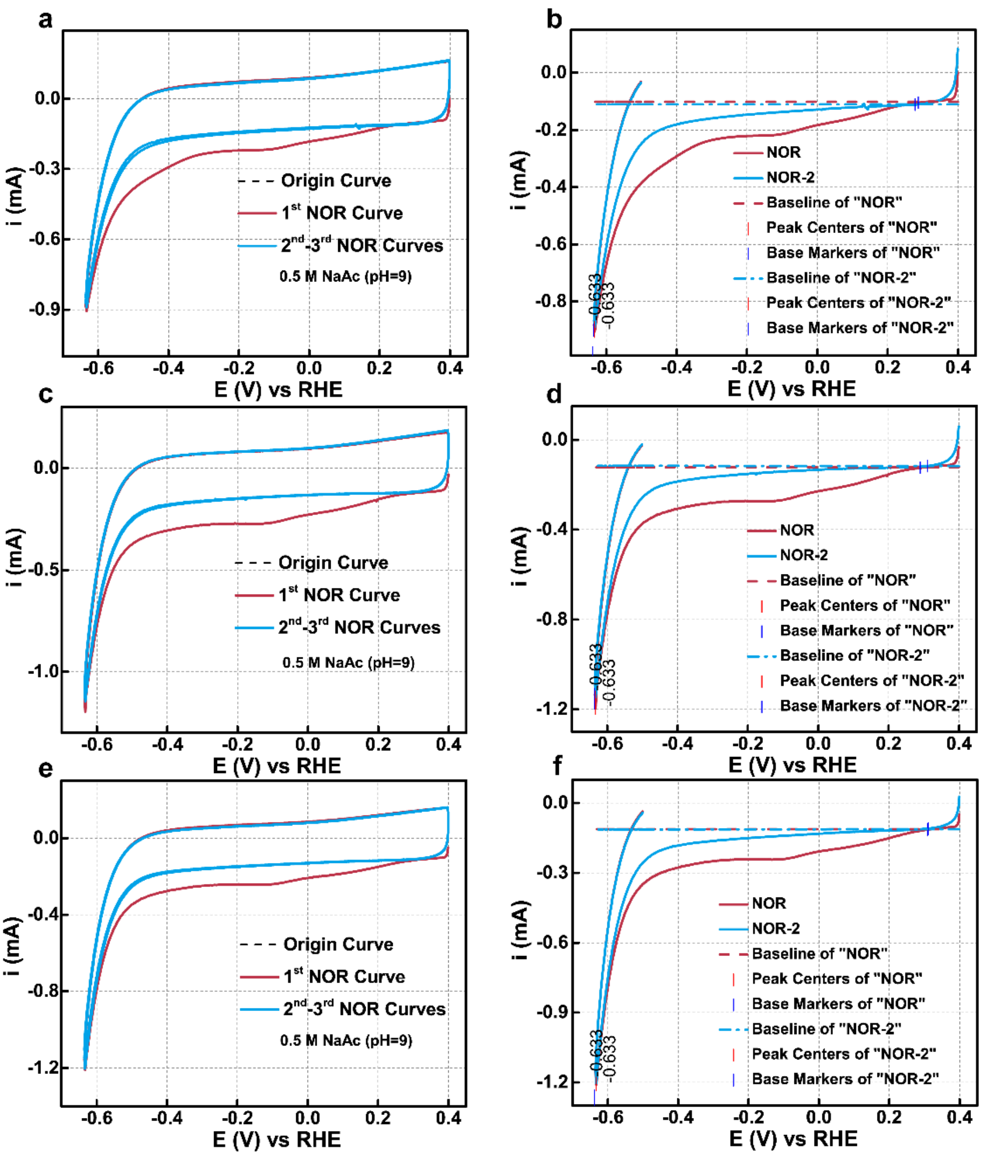

Supplementary Fig. 34 The NOR repeatability test and integral electricity calculation of FeNC by NAEM method in the 0.5 M NaAc solution (pH = 9). The resistance is about 20 Ω.

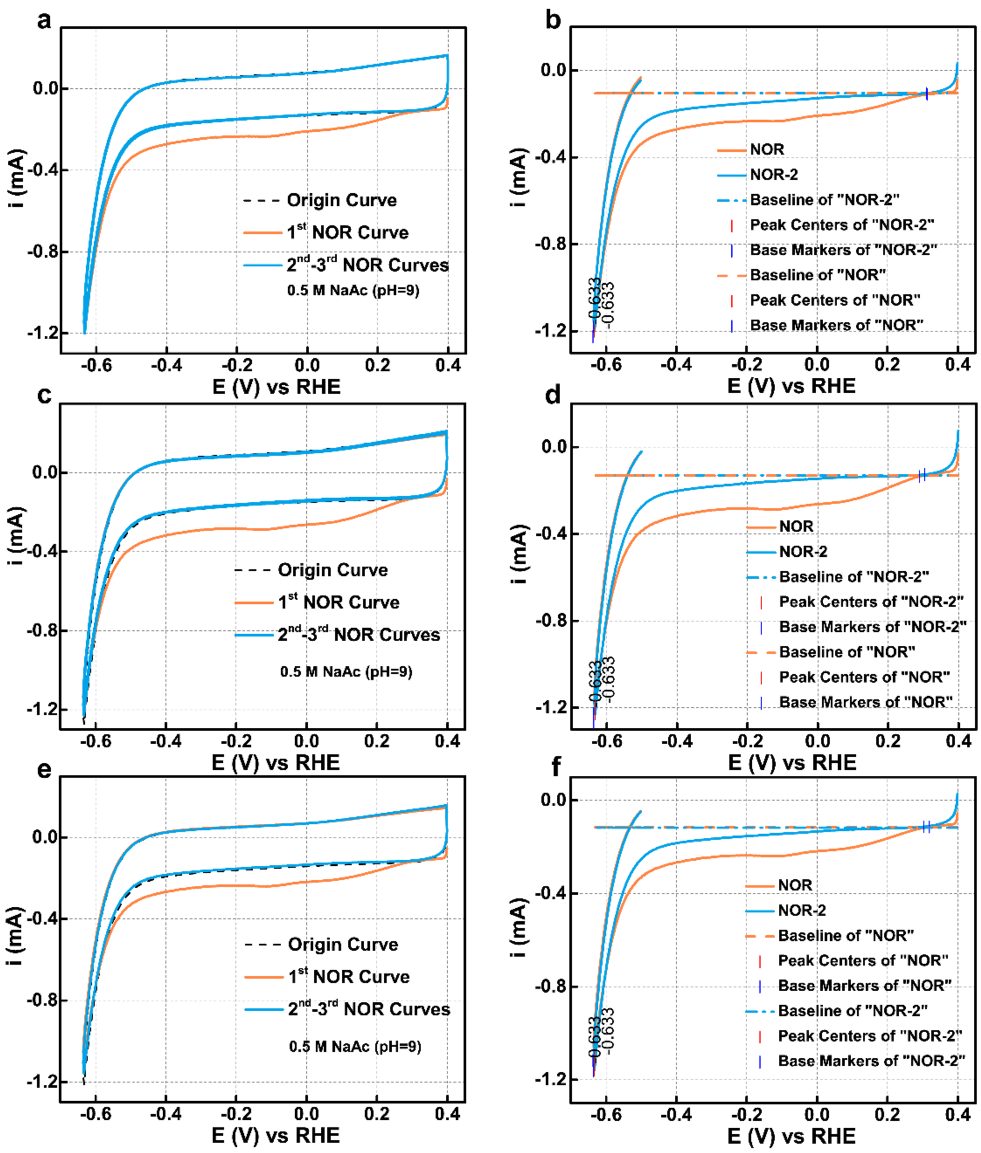

Supplementary Fig. 35 The NOR repeatability test and integral electricity calculation of FeNC by ASNPM method in the 0.5 M NaAc solution (pH = 9). The resistance is about 20 Ω.

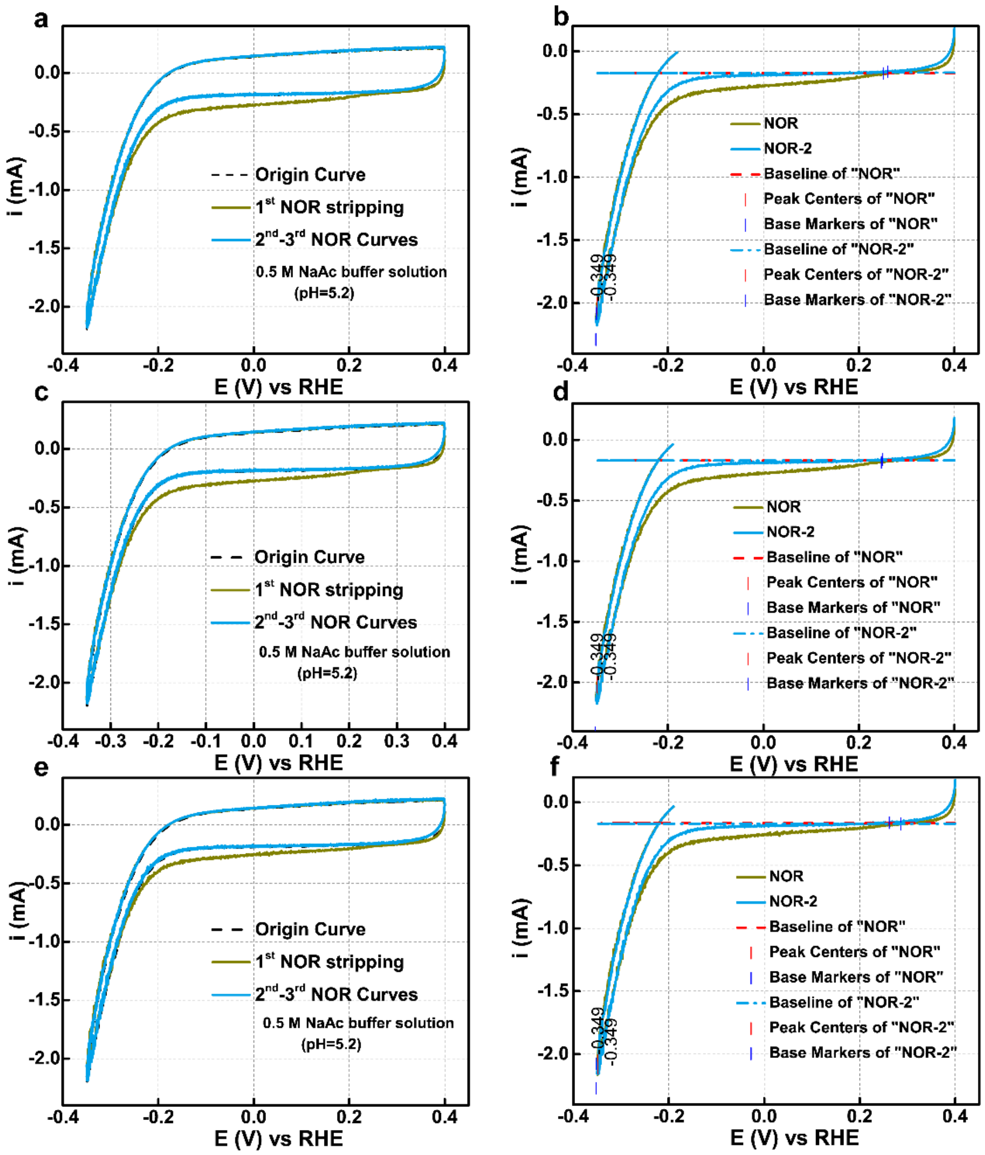

Supplementary Fig. 36 The NOR repeatability test and integral electricity calculation of CoNC by NPM method in the 0.5 M NaAc buffer solution (pH = 5.2). The resistance is about 20 Ω.

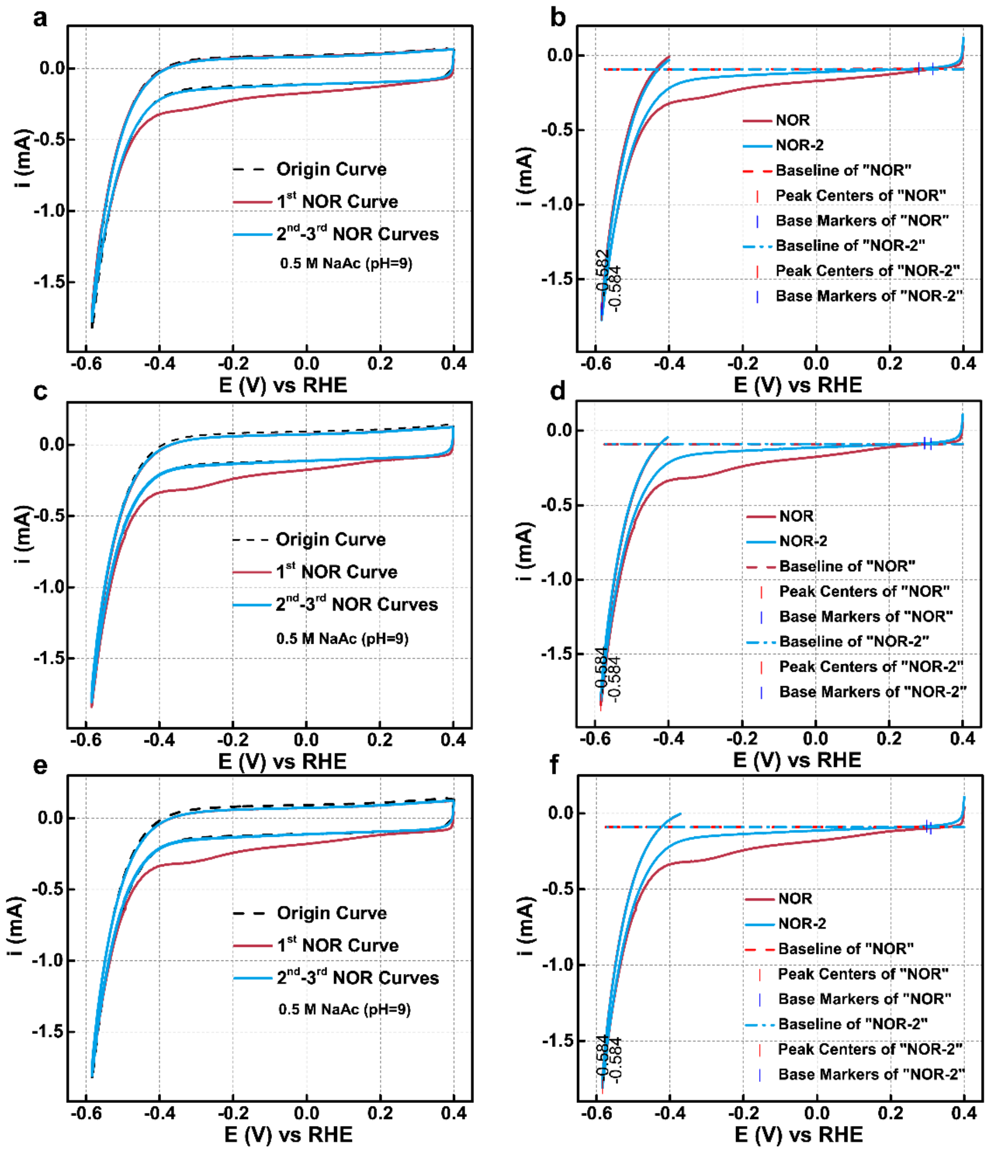

Supplementary Fig. 37 The NOR repeatability test and integral electricity calculation of CoNC by NAEM method in the 0.5 M NaAc solution (pH = 9). The resistance is about 20 Ω.

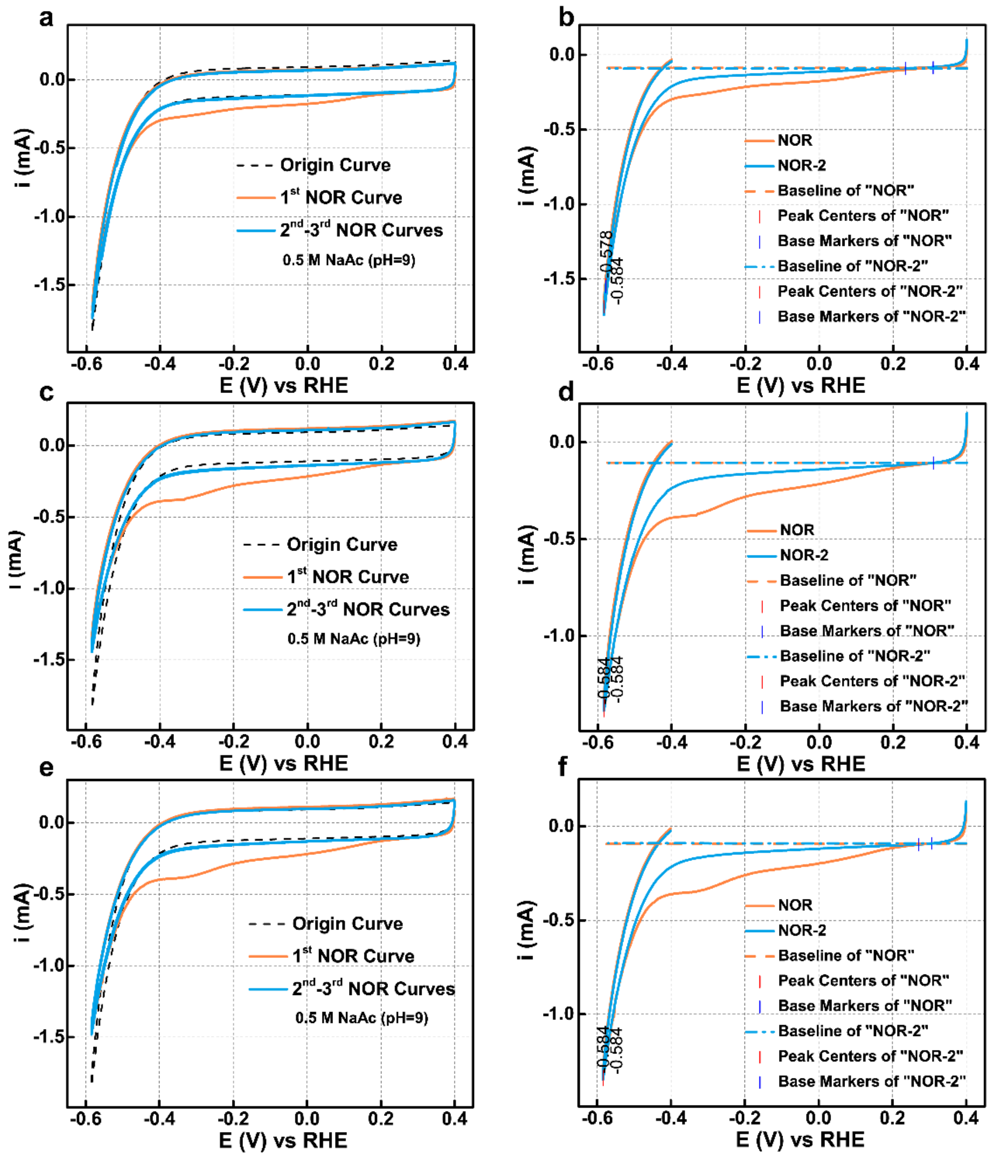

Supplementary Fig. 38 The NOR repeatability test and integral electricity calculation of CoNC by AANPM method in the 0.5 M NaAc solution (pH = 9). The resistance is about 20 Ω.

Discussion on the negative shift of the lower limit potential and the influence of HER

The lower potential limit was selected to ensure complete reduction of adsorbed NO species. As demonstrated in **Supplementary Fig. 38a** (traditional NPM method, lower limit: -0.3 V *vs.* RHE), non-overlapping 1st and 2nd NOR polarization curves at -0.3 V indicate incomplete NO reduction. Extending the potential to -0.6 V *vs.* RHE (**Supplementary Fig. 38b**) achieves full reduction, evidenced by overlapping NOR profiles in subsequent cycles. Although hydrogen evolution (HER) occurs at this

potential, two critical points are emphasized:

(i) The charge attributable exclusively to NOR is obtained by subtracting the 2nd cycle (background, post-NO purge) from the 1st cycle (NOR activity), effectively eliminating HER contributions.

(ii) Reproducible NOR charge integrals across multiple cycles (**Supplementary Figs. S32–S37**) confirm the absence of structural degradation. The consistent NOR activity following repeated polarization to -0.6 V *vs.* RHE underscores the robustness of the M-N_4 sites under these conditions.

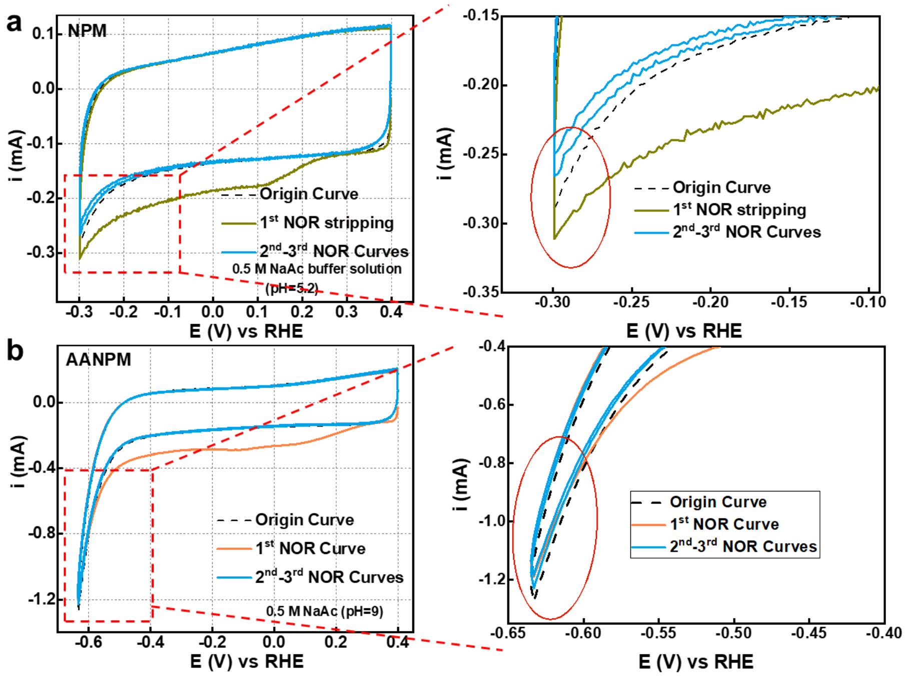

Supplementary Fig. 39 (a) The NOR test and the corresponding lower limit potential amplification region of FeNC in (a) NPM and (b) AANPM processes.

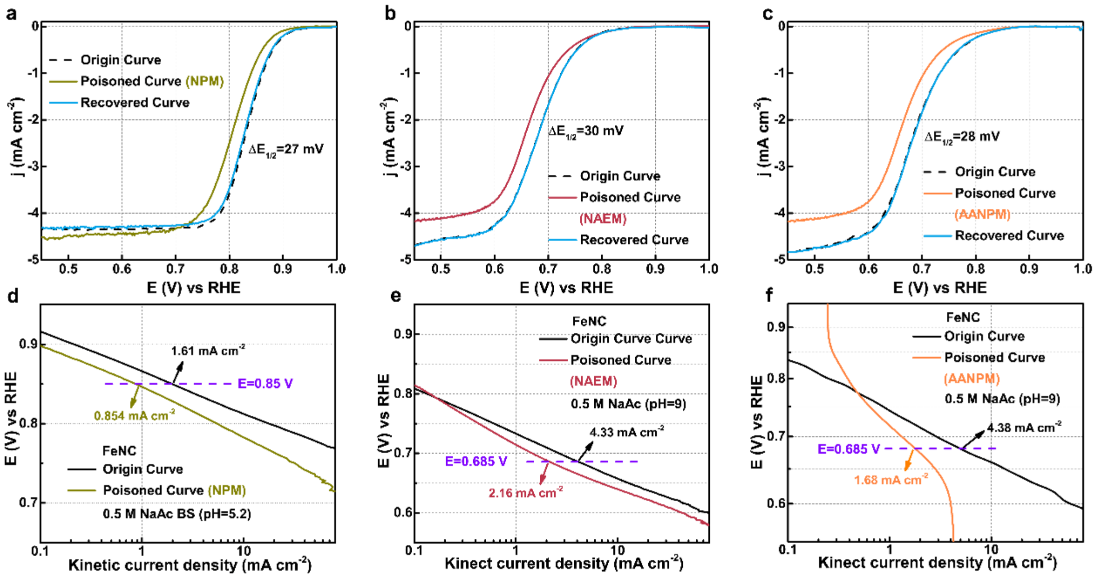

Supplementary Fig. 40 The ORR test curves and dynamic current density calculation of FeNC under different methods. The resistance is about 20 Ω.

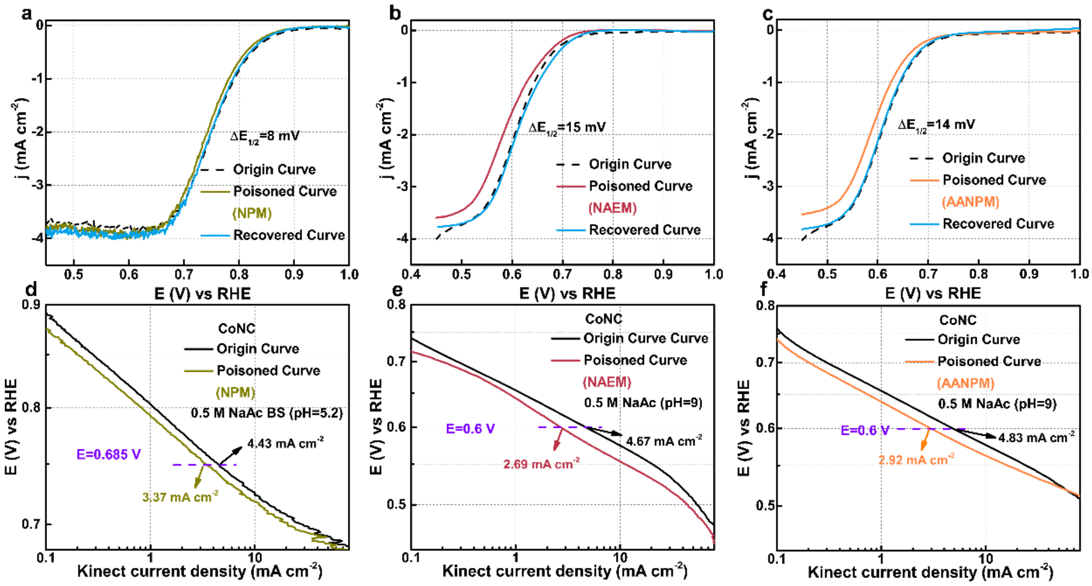

Supplementary Fig. 41 The ORR test curves and kinetic current density calculation of CoNC under different methods. The resistance is about 20 Ω.

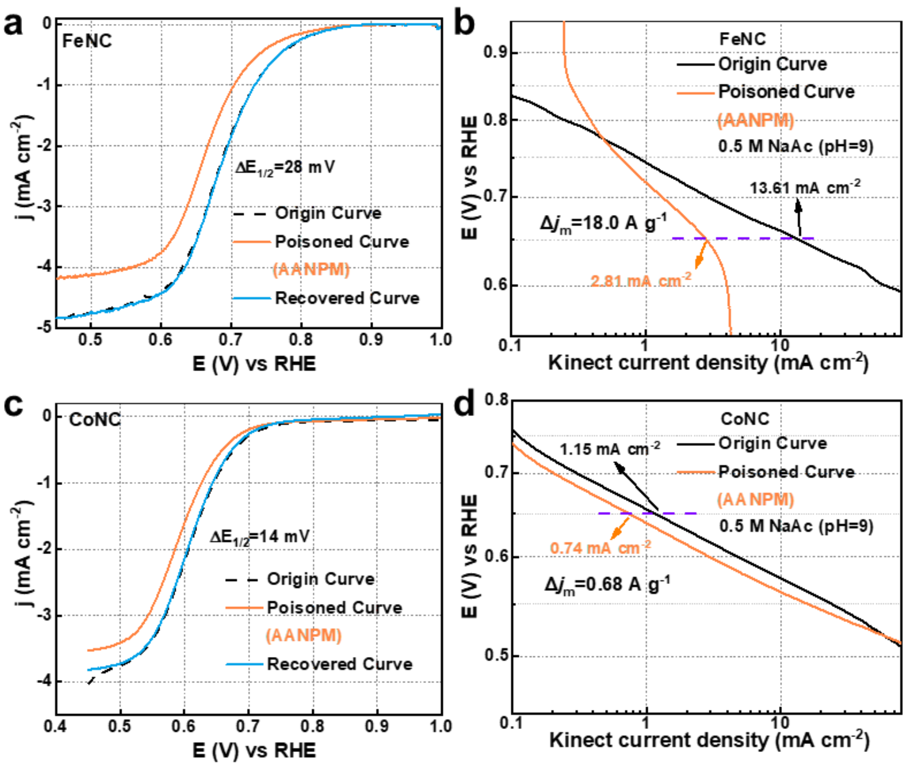

Supplementary Fig. 42 The ORR test curves and kinetic current density at 0.65 V of (a, b) FeNC and (c, d) CoNC by AANPM method. The resistance is about 20 Ω.

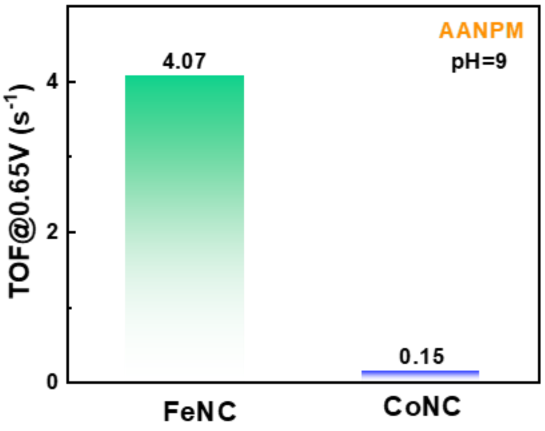

Supplementary Fig. 43 The TOF values at 0.65 V of FeNC and CoNC.

As shown in **Fig. 7f**, the AANPM method yields comparable SD values for FeNC and CoNC (~2.8 × 10^{19} sites g^{-1}). However, their ORR activities differ markedly. By combining the changes in mass activity at 0.65 V (Δj_m@0.65V) with the SD values (**Supplementary Fig. 42**), we calculated the turnover frequency (TOF) for each catalyst, as shown in **Supplementary Fig. 43**. The results reveal a two-order-of-magnitude difference in TOF (FeNC: 4.07 s^{-1}; CoNC: 0.15 s^{-1} at 0.65 V *vs.* RHE), primarily reflecting the intrinsic activity difference between Fe and Co sites. The AANPM method cannot account for site heterogeneity, particularly in MNC catalysts where metal centers may exist in diverse coordination environments. In this case, the activity gap between FeNC and CoNC arises mainly from differences in their electronic structures rather than coordination geometries. This consistency supports the reliability of our site quantification and reinforces that the observed activity difference originates from TOF variations rather than discrepancies in SD. Therefore, while the AANPM method effectively quantifies accessible metal sites capable of binding the probe molecule, it does not distinguish between sites with different catalytic activities.

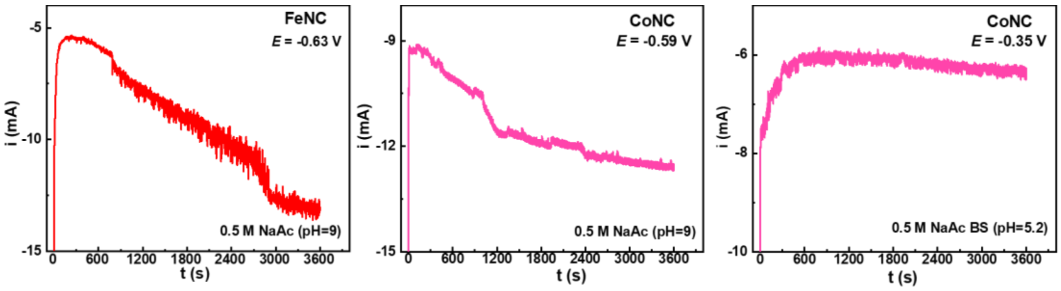

Supplementary Fig. 44 The constant potential test of NOR test for FeNC and CoNC catalysts. The resistance is about 20 Ω.

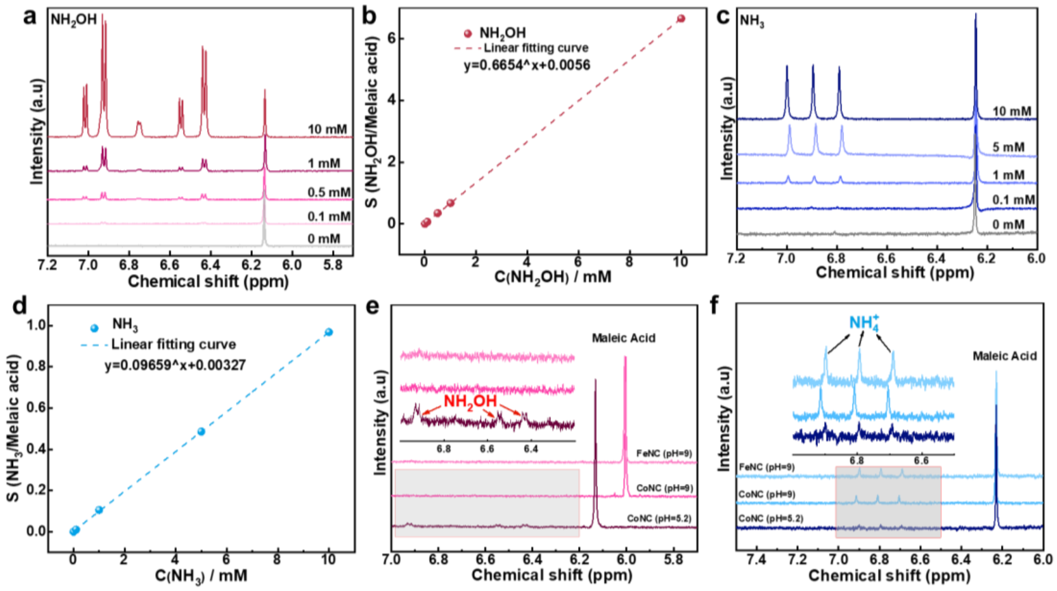

Supplementary Fig. 45 (a)-(d) The NMR spectra and standard curves of standard samples for NH_3 and NH_2OH. (e)-(f) The analysis of NOR products of FeNC and CoNC.

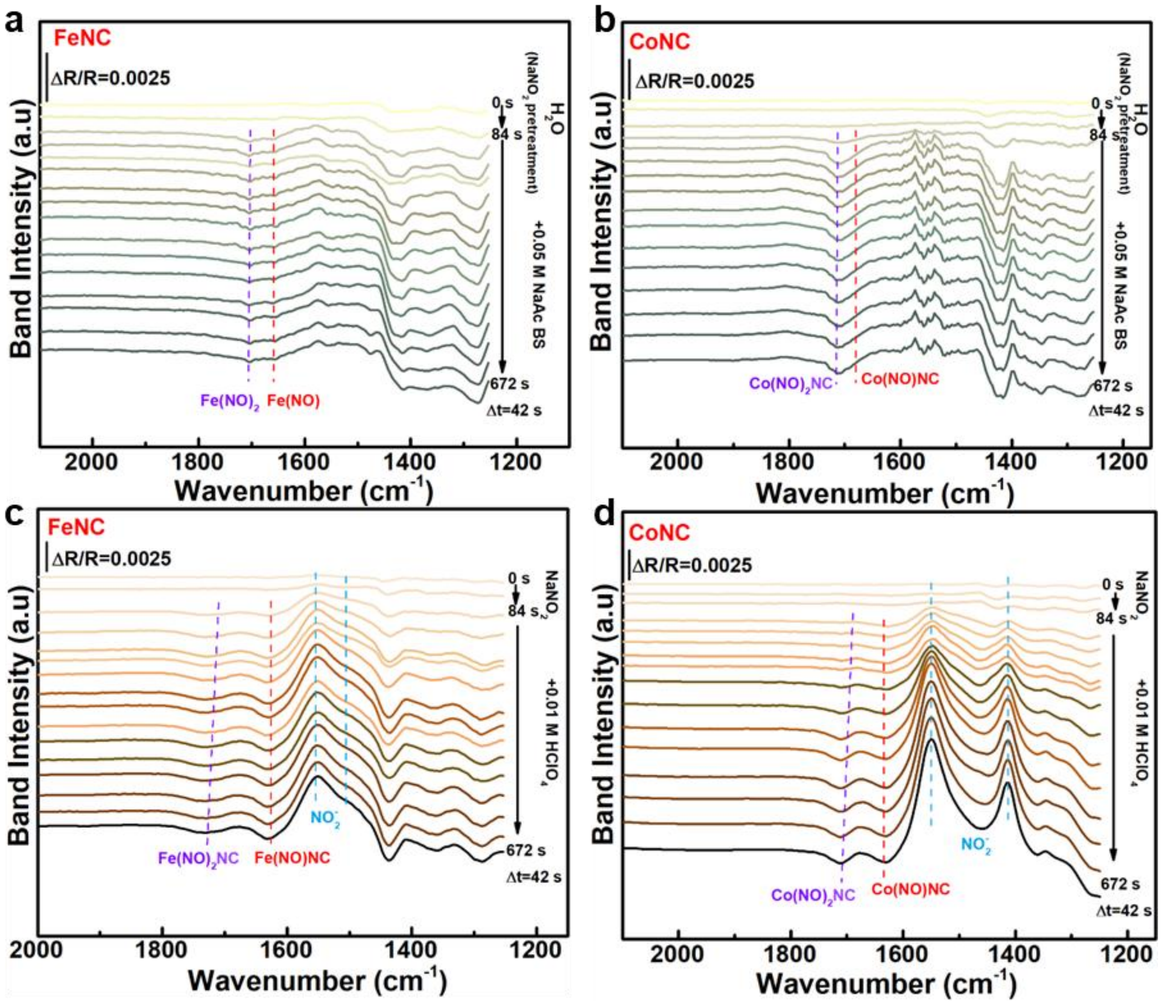

Supplementary Fig. 46 The gra-based *in-situ* ATR-FTIR of NO adsorption process on FeNC and CoNC catalysts. (a)-(b) NPM process; (c)-(d) AANPM process. The NO adsorption wavenumber is affected by H_2O, and it is difficult to quantitatively analyze the peak area. Therefore, isotope experiments are needed to eliminate the interference of H_2O.

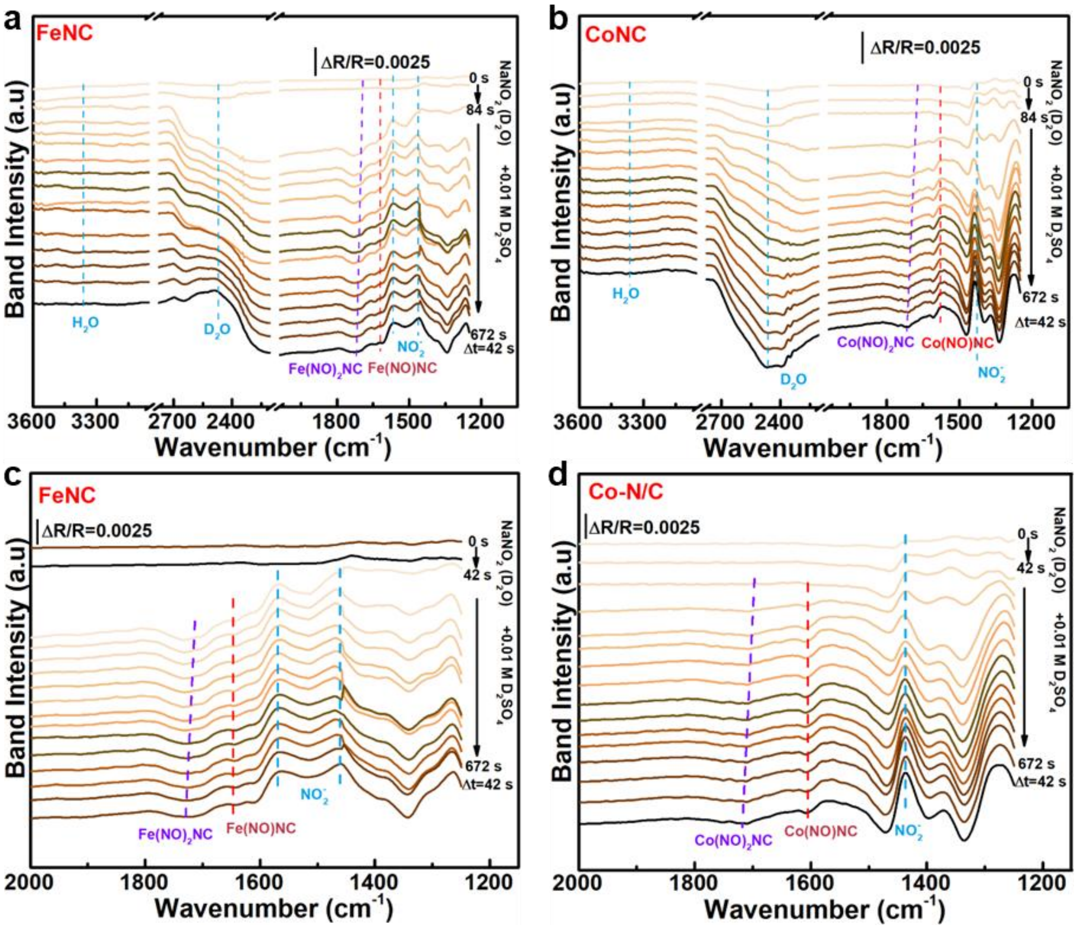

Supplementary Fig. 47 The gra-based *in-situ* ATR-FTIR of NO adsorption process on FeNC and CoNC catalysts by AANPM method with isotope experiment, 0.125 M $NaNO_2$ (dissolved in D_2O) +0.01 M D_2SO_4. (c)-(d) was the detail drawing of (a)-(b).

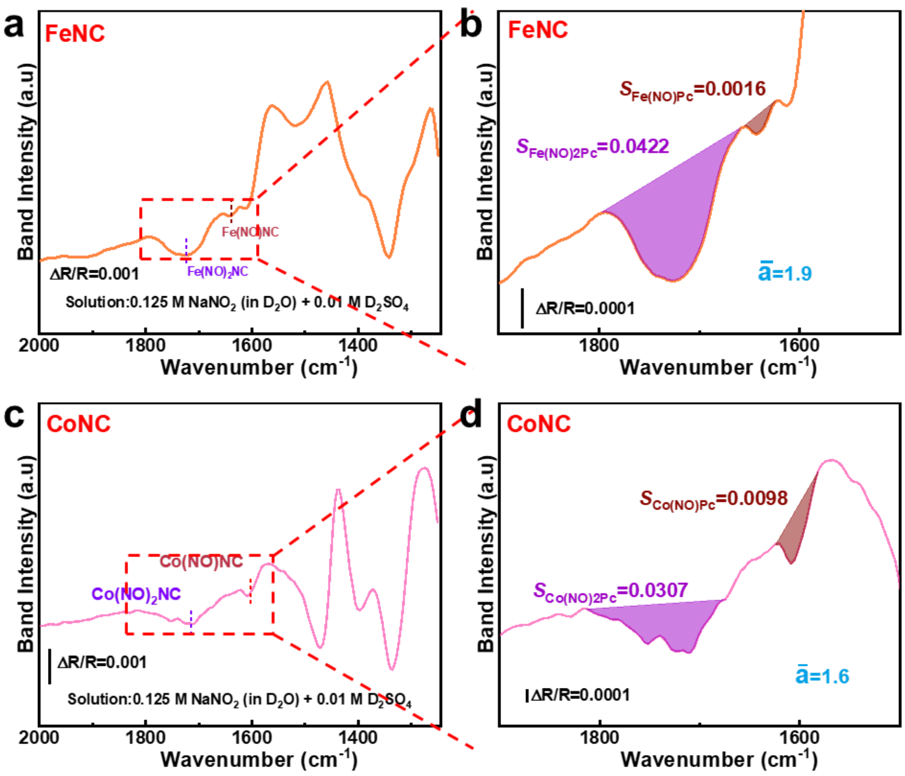

Supplementary Fig. 48 (a) NO_{ad} spectra of FeNC and (b) the corresponding spectral peak intergral area of $S_{Fe(NO)2Pc}$ and $S_{Fe(NO)Pc}$. (c) NO_{ad} spectra of CoNC and (d) the corresponding spectral peak intergral area of $S_{Co(NO)2Pc}$ and $S_{Co(NO)Pc}$.

To ensure robust quantification, all spectra were subjected to rigorous third-order polynomial baseline correction using OMNIC's advanced tools, applied consistently to both model complexes (FePc/CoPc) and pyrolyzed catalysts (FeNC/CoNC). Despite peak broadening, the 1710 cm^{-1} and 1600 cm^{-1} bands were resolved via peak-fitting deconvolution. Importantly, quantification was based on relative peak area ratios rather than absolute intensities.

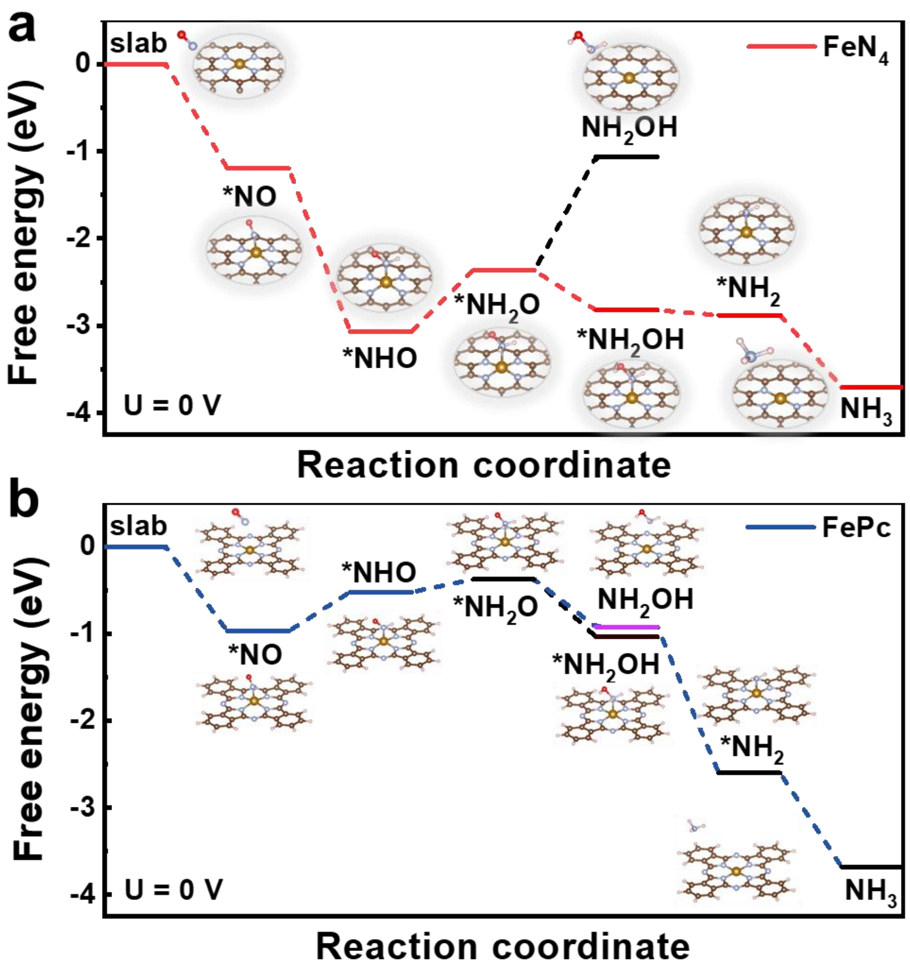

Supplementary Fig. 49 Free energy diagram of acidic ORR on Fe center of (a) FePc and (b) FeN_4 site at U = 0 V.

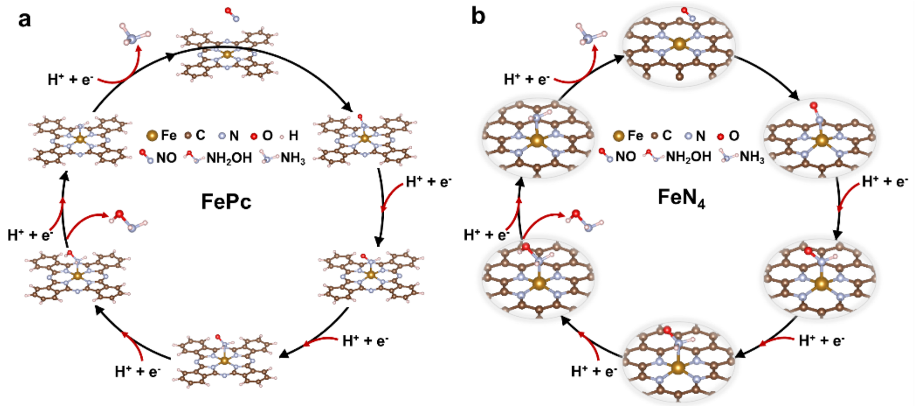

Supplementary Fig. 50 The adsorption structures for *NO, *NHO, *NH_2O, *NH_2OH, *NH_2 in ORR process of the established configurations at Fe center of (a) FePc and (b) FeN_4 site.

Explicit DFT simulations of NO adsorption configurations and electron transfer pathways on MNC sites would strengthen the mechanistic interpretation of our spectral

data and reaction pathways. In our supplementary DFT analysis, we have mapped the free-energy landscapes for NO reduction on both FeN_4 and FePc sites, revealing distinct thermodynamic bottlenecks. On FeN_4, the thermodynamic rate-determining step (RDS) is $*NHO + H+ + e^- \rightarrow *NH_2O$. On FePc, the RDS is $*NO + H+ + e^- \rightarrow *NHO$. Importantly, the calculations also indicate a stronger thermodynamic tendency for $3e^-$ reduction to NH_2OH on FePc than on FeN4. However, we recognize that our present model does not explicitly account for solvent pH effects, which critically influence protonation kinetics. This divergence from the $5e^-$ pathway predicted by standard DFT (at U = 0 V *vs.* SHE) underscores the role of acidic microenvironments in lowering kinetic barriers for proton-coupled steps, thereby favoring the $3e^-$ pathway to NH_2OH.

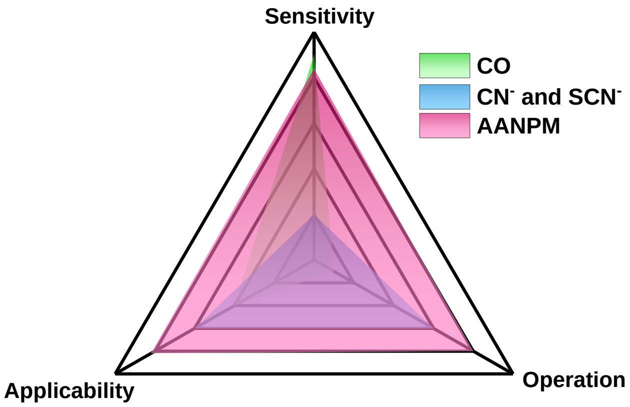

Supplementary Fig. 51 Several probe molecular methods for quantitative comparison of MNC catalyst active site density radar map.

The comparison in terms of sensitivity, operational complexity, and applicability scenarios of different quantitative methods such as CO, CN^-, and SCN^- poisoning methods

(i) Sensitivity

AANPM exhibits excellent sensitivity, comparable to CO pulse chemisorption (the gold standard for quantifying active sites in MNC catalysts) [*Nat. Commun. 2015, 6, 8618*]; and importantly, it eliminates the need for ultralow-temperature conditions (193 K). Our validation experiments on FeNC and CoNC confirm that the site densities obtained from AANPM closely match those derived from CO chemisorption. CN^- and SCN^- poisoning, while sensitive, often suffer from incomplete site blocking due to competitive adsorption or side reactions (ligand exchange with SCN^-), leading to potential underestimation.

(ii) Operational complexity

AANPM is significantly simpler than CO-adsorption, which requires cryogenic temperatures (< −80 °C) and ultra-high-purity gas handling. In contrast to CN^- poisoning (requiring strict anaerobic conditions due to CN^-'s oxygen sensitivity and extreme toxicity), AANPM employs benign reagents under ambient conditions. Unlike SCN^- poisoning, which is complicated by pH-dependent equilibria and poor reproducibility, AANPM operates reliably across a wide pH range (3–11).

(iii) Applicability scenarios

AANPM is broadly applicable to FeNC and CoNC (validated herein) and is, in principle, extendable to other MNCs (e.g., MnNC, ZnNC), although further studies are needed to account for possible differences in binding energetics (see limitations below). Unlike CO adsorption, which cannot distinguish between metal sites (e.g., Fe vs. Co) in bimetallic catalysts, AANPM's selectivity can be tuned through reagent design. CN^- and SCN^- are restricted to aqueous systems and may corrode certain substrates (e.g., SCN^- attacks Cu-based catalysts)

However, currently validated only for FeNC and CoNC; extension to other MNCs (e.g., MnNC, ZnNC) requires further study, as their binding energetics may differ."

Supplementary Table 1 The ICP-MS result of molecular catalyst and FeNC, CoNC

Samples	The mass fractions of Fe by ICP (%)	The mass fractions of Co by ICP (%)
FePc-KJ	1.7576	—
FeNC	1.4567	—
$CoPc_{ag}$-KJ	—	2.6578
CoPc-KJ	—	1.2927
CoNC	—	2.0284

Supplementary Table 2 Curvefit Parametes[a] for Co and Fe K-edge EXAFS for J-1Fe and Co standards

Samples	Paths	N	R (Å)	σ^2×10^3 ($Å^2$)	ΔE_0 (eV)
CoNC	Co-N	4.72±1.11[c]	1.88	9.32[d]	-14.32
FeNC	Fe-N	4.2±0.8	2.01	13.7	0.5
